# Infection prevention and control policies in hospitals and prevalence of highly resistant microorganisms: an international comparative study

**DOI:** 10.1186/s13756-022-01165-0

**Published:** 2022-12-06

**Authors:** Manon D. van Dijk, Anne F. Voor in ’t holt, Emine Alp, Markus Hell, Nicola Petrosillo, Elisabeth Presterl, Athanasios Tsakris, Juliëtte A. Severin, Margreet C. Vos, ESCMID Study Group for Nosocomial Infections (ESGNI)

**Affiliations:** 1 Department of Medical Microbiology and Infectious Diseases, Erasmus MC University Medical Centre Rotterdam, P.O. Box 2040, 3000 CA, Rotterdam, The Netherlands; 2 Department of Infectious Diseases and Clinical Microbiology, Medical Faculty, Erciyes University, Kayseri, Turkey; 3 Department of Infectious Diseases and Clinical Microbiology, Medical Faculty, Ankara Yıldırım Beyazıt University, Ankara, Turkey; 4 Department of Clinical Microbiology and Infection Control, MEDILAB-Academic Teaching Laboratories, Paracelsus Medical University, Salzburg, Austria; 5 Teaching Hospital, Kardinal Schwarzenberg Klinikum, Paracelsus Medical University, Schwarzach, Austria; 6 Clinical and Research Department for Infectious Diseases, National Institute for Infectious Diseases “Lazzaro Spallanzani”, Rome, Italy; 7 Department of Infection Control, University Hospital Campus Bio-Medico, Rome, Italy; 8 Department of Infection Control and Hospital Epidemiology, Medical University of Vienna, Vienna, Austria; 9 Department of Microbiology, Medical School, University of Athens, Athens, Greece; 10 ESCMID Study Group for Nosocomial Infections (ESGNI), ESCMID Study Group for Nosocomial Infections (ESGNI), Basel, Switzerland

**Keywords:** Policy, Carbapenemase-producing *Klebsiella pneumoniae*, Prevalence, Carbapenemase-producing *Pseudomonas aeruginosa*, Vancomycin-resistant *Enterococcus faecium*, Infection control, Surveys and Questionnaires, Drug Resistance, Microbial, Hospitals

## Abstract

**Background:**

There are differences in infection prevention and control (IPC) policies to prevent transmission of highly resistant microorganisms (HRMO). The aim of this study is to give an overview of the IPC policy of six European hospitals and their HRMO prevalence, to compare the IPC policies of these hospitals with international guidelines, and to investigate the hospitals’ adherence to their own IPC policy.

**Methods:**

The participating hospitals were located in Salzburg (Austria), Vienna (Austria), Kayseri (Turkey), Piraeus (Greece), Rome (Italy) and Rotterdam (The Netherlands). Data were collected via an online survey. Questions were aimed at prevalence rates in the years 2014, 2015, 2016 of carbapenemase-producing *Klebsiella pneumoniae* (CPK), carbapenemase-producing *Pseudomonas aeruginosa* (CPPA), vancomycin-resistant *Enterococcus faecium* (VRE) and hospitals’ IPC policies of 2017. Implemented IPC measures (i.e. with a self-reported adherence of > 90%) were counted (26 points maximal).

**Results:**

The self-reported prevalence of CPK per year was low in the Austrian and Dutch hospitals and high in the Turkish and Greek hospitals. CPPA was highly prevalent in the Turkish hospital only, while the prevalence of VRE in four hospitals, except the Austrian hospitals which reported lower prevalence numbers, was more evenly distributed. The Dutch hospital had implemented the most IPC measures (n = 21), the Turkish and Greek hospitals the least (n = 14 and 7, respectively).

**Conclusion:**

Hospitals with the highest self-reported prevalence of CPK and CPPA reported the least implemented IPC measures. Also, hospitals with a higher prevalence often reported a lower adherence to own IPC policy.

**Supplementary Information:**

The online version contains supplementary material available at 10.1186/s13756-022-01165-0.

## Background

In recent years, there has been a worldwide increase of highly resistant microorganisms (HRMO) in hospitalized patients [1, 2]. Organisations like the World Health Organisation (WHO), the Centres for Disease Control and prevention (CDC), European Centre for Disease Prevention and Control (ECDC) and the European Society of Clinical Microbiology and Infectious Diseases (ESCMID) provide evidence-based guidelines for the management, prevention and transmission of HRMO in hospitals [3–5]. National infection prevention and control (IPC) HRMO policies are often largely based on these international guidelines, but also on expert opinion.

As a result, IPC HRMO policies could differ between and within countries [6, 7]. The differences in policy are mainly based on passive or active identification of HRMO in patients. Hospitals could primarily rely on clinical cultures to detect HRMO [8] or they could focus on early identification of colonized patients, by actively screening patients upon admission. Through early identification of colonized patients, IPC measures (e.g. isolation, contact investigation) can be installed sooner [1, 9–11]. Depending on the HRMO involved, and in case of transmission, hospitals can intensify IPC measures [1, 5, 10]. Early identification is favourable, as it will reduce transmission to patients and to the hospital environment. However, some hospitals do not perform active screening of patients, for example, due to a lack of resources. IPC policies may also differ in isolation practices, use of personal protective equipment (PPE), electronic labelling, microbiological methods, and cleaning and disinfection [12]. We aim to give an overview of the IPC policy of six European hospitals and their HRMO prevalence. Second, we aim to compare the HRMO IPC policies of six European hospitals with international IPC guidelines, and third, we aim to investigate the respective hospitals’ adherence to their own IPC policy.

## Methods

### Study design

This international observational comparative study retrospectively collected data from January 2014 until January 2017. The study collected data on HRMO prevalence and IPC policy and measures.

### Study population

Six hospitals from five different countries participated in this study: (1) Erasmus MC University Medical Centre in Rotterdam, The Netherlands (EMC), (2) Erciyes University in Kayseri, Turkey (ERU), (3) Kardinal Schwarzenberg Klinikum in Salzburg, Austria (KSK), (4) National Institute for Infectious Diseases ‘Lazzaro Spallanzani’ in Rome, Italy (INMI), (5) Tzaneio General Hospital in Piraeus, Greece (TGH), and (6) Vienna General Hospital in Vienna, Austria (VGS).

The five countries were selected based on their prevalence rates of HRMO as reported in the ECDC maps of 2015 [13], and for Turkey as reported in the WHO Central Asian and Eastern European Surveillance of Antimicrobial Resistance (CAESAR) of 2016 [14], in such a way that an equilibrium between low (< 1% and 1-<5%), medium (5-<10% and 10-<25%) and high (25-<50%, 50-<75% and > = 75%) prevalence countries was ensured. Selection of hospitals was done by and from members of the ESCMID Study Group for Nosocomial Infections (ESGNI).

### Data collection

This study focussed on the following HRMO: (1) Carbapenemase-producing *Klebsiella pneumoniae* (CPK), (2) Carbapenemase-producing *Pseudomonas aeruginosa* (CPPA), and (3) Vancomycin-resistant *Enterococcus faecium* (VRE, only VanA and/or VanB). Carbapenemase genes included: *bla*_KPC_ (class A), *bla*_NDM_, *bla*_VIM_, *bla*_IMP_ (class B), and *bla*_OXA-48_ (class D) [15]. When microbial laboratories of the participating hospitals could only provide information about the susceptibility pattern of microorganisms and did not investigate the underlying mechanisms of resistance, we used the phenotypical data and included carbapenem-resistant *K. pneumoniae*, carbapenem-resistant *P. aeruginosa* and vancomycin-resistant *E. faecium*.

From 18 July 2017 until 24 September 2017 we asked our contacts from the participating hospitals to fill in an online survey with detailed information on their IPC policy of 2017, and prevalence rates of 2014, 2015, 2016 of the included HRMO (Additional file 1). For ERU and KSK the contacts themselves filled in the survey, for the EMC an IPC specialist filled it in, and for INMI, TGH and VGS a postdoctoral researcher filled in the survey. Hospitals also had to indicate on a 5-point Likert scale (e.g. (1) No idea, (2) Rarely / never (< 10%), (3) Sometimes (10–49%), (4) Usually (50–90%), (5) All the time (> 90%)) their adherence to their own IPC policy, per measure and per HRMO.

From each hospital, aggregated HRMO data from all patients was collected and sent in, without being retraceable to individual patients. Inclusion of patients was irrespective of sample site, but each patient was only counted once per year for each HRMO. Cystic fibrosis patients were excluded as these patients are known to carry HRMO, especially *Pseudomonas spp.* [16].

The survey was pilot tested by two medical microbiologists, an IPC expert, and the manager of the diagnostics department of the Erasmus MC, and adjusted accordingly before it was sent to the participating hospitals.

### Analysis

The IPC policies of the six participating hospitals were compared to international IPC guidelines of the ESCMID [5], the WHO [17–20], the ECDC [21] and CDC [22–24] (Additional file 2). The following uniform definitions were used for comparison; primary case: ‘The first indicated patient in whom a clinical or screening sample was unexpectedly positive for a certain HRMO’. A secondary case: ‘A patient, linked in time and place, and with the same HRMO as the primary/index case’. An outbreak: ‘Two or more similar HRMO cases linked in time and place’. We also scored the hospitals based on the number of implemented IPC measures. A hospital got one point for each implemented IPC measure, but only when they indicated to adhere to the IPC measure for more than 90% (all the time).

## Results

### The participating hospitals

Characteristics of the participating hospitals are displayed in Table 1. During the study period, EMC, VGS and TGH tested for *bla*_IMP_, *bla*_VIM_, *bla*_OXA−48_, *bla*_KPC_, *bla*_NDM_; EMC and VGS additionally tested for *VanA* and *VanB.* In 2017, KSK started testing for carbapenemase genes *bla*_OXA−48_, *bla*_KPC_, and *bla*_NDM_.

**Table 1:** Characteristics of the six participating hospitals

		EMC (Netherlands)	KSK (Austria)	VGS (Austria)	INMI (Italy)	ERU (Turkey)	TGH (Greece)	Median
No. of hospital beds		1184	500	1914	157	1000	452	750
No. of single-patient rooms (%)		211 (18)	10 (2)	148 (8)	54 (34)	278 (28)	8 (2)	101 (13)
Average number of patients in a room		2	3	2.5	1.5	3	4	3
No. of infection control practitioners employed (on average present per day)		8 (4)	1 (1)	6 (5)	5 (1)	6 (6)	2 (2)	6 (3)
No. of physicians specialized in infectious diseases* employed (on average present per day)		4 (1)	1 (1)	12 (1)	60 (40)	5 (5)	10 (8)	8 (7)
Clinical hospital admissions	2014	36,976	29,571	105,930	3037	163,712	20,804	33,274
	2015	36,811	29,695	106,869	2906	169,774	20,246	33,253
	2016	37,858	29,357	114,030	3090	173,482	20,486	33,608
Clinical hospital admission days	2014	288,865	143,651	524,087	43,785	466,275	87,168	216,258
	2015	289,124	142,084	518,262	44,306	470,543	83,546	215,604
	2016	288,734	141,569	513,926	49,620	453,503	80,449	215,152

*Medical microbiologists and infectious disease specialists. Abbreviations; No.: number. EMC: Erasmus MC University Medical Centre in Rotterdam, The Netherlands. KSK: Kardinal Schwarzenberg Klinikum in Salzburg, Austria. VGS: Vienna General Hospital in Vienna, Austria. INMI: National Institute for Infectious Diseases ‘Lazzaro Spallanzani’ in Rome, Italy. ERU: Erciyes University in Kayseri, Turkey. TGH: Tzaneio General Hospital in Piraeus, Greece

### General IPC measures

To identify if patients had an increased risk of HRMO, four hospitals (66.7%) performed a risk-based screening upon hospital admission (Additional file 2). KSK and TGH (33.3%) only performed a risk-based screening upon hospital admission when there were known indications that they might have an increased risk of HRMO. Furthermore, only AKH and TGH did not isolate patients when triage showed that the patient had an increased risk of HRMO. Additionally, TGH did not isolate patients who already had an isolation label in their electronic health record (Additional file 2). Furthermore, the specific definitions that hospitals used for a primary case, secondary case and an outbreak are described in the Additional file 3.

### CPK specific IPC measures

The hospitals with the lowest median self-reported prevalence of CPK (EMC, KSK and VGS) were all situated in countries that were indicated by the ECDC and WHO as low prevalence countries (Table 2a, Additional file 4). The same applied for the hospitals with the highest median self-reported prevalence (ERU, INMI and TGH).

**Table 2:** **a.** Comparing IPC policies on CPK

	EMC (Netherlands)	KSK (Austria)	VGS (Austria)	ERU (Turkey)	INMI (Italy)	TGH (Greece)
**Prevalence according to ECDC maps 2015 and CAESAR 2016 (13, 14)**	0.2%	0.8%	0.8%	30%	33.5%	62%
	Low	Low	Low	High	High	High
**Median self-reported prevalence of HRMO per hospital per year, regardless of sample site (range 2014–2016)**	4 (2–5)	0 (0–0)	3 (2–17)	382 (190–399)	66 (34–69)	178 (156–191)
Targeted screening^§^						
Primary case^¥^ - targeted screening on hospitalized patients	Yes (> 90%)*	Yes (> 90%)	Yes (> 90%)	*Yes (> 90%)*	Yes (50–90%)	*Yes (10–49%)*
Primary case - targeted screening on discharged patients	*Yes (> 90%)*	No	No	*Yes (50–90%)*	No	No
Secondary case – targeted screening on hospitalized patients	*Yes (> 90%)*	Yes (> 90%)	Yes (> 90%)	*Yes (50–90%)*	Yes (> 90%)	Yes (50–90%)
Secondary case – targeted screening on discharged patients	*Yes (> 90%)*	No	No	*Yes (50–90%)*	No	No
Outbreak – targeted screening on hospitalized patients	*Yes (> 90%)*	Yes (> 90%)	Yes (> 90%)	*Yes (50–90%)*	Yes (> 90%)	Yes (50–90%)
Outbreak – targeted screening on discharged patients	*Yes (> 90%)*	No	No	*Yes (50–90%)*	*Yes (50–90%)*	No
Labelling						
Isolation label for CPK-positive patients	Yes (> 90%)	*Yes, but without HRMO specification (50–90%)*	Yes (50–90%)	Yes (> 90%)	Yes (> 90%)	Yes (> 90%)
Number of negative cultures before lifting label	6, during one year	N.D.	*Upon discharge (but stays archived digitally)*	3, one week apart	3	3
Isolation measures						
Isolation in multi-bedroom with blocking of the beds	No	*No*	Yes (50–90%)	Yes (50–90%)	Yes (> 90%)	No
Isolation in single bedroom without anteroom	Yes (> 90%)	*No*	Yes (> 90%)	Yes (50–90%)	No	Yes (50–90%)
Isolation in single bedroom with anteroom	No	*No*	No	Yes (10–49%)	Yes (> 90%)	No
Personal protective equipment						
Non-sterile gloves	Yes (> 90%)	Yes (> 90%)	*Yes (> 90%)*	*Yes (> 90%)*	Yes (> 90%)	Yes (50–90%)
Disposable gowns	Yes (> 90%)	Yes (> 90%)	*Yes (> 90%)*	*Yes (> 90%)*	Yes (> 90%)	Yes (50–90%)
Caps	No	No	*Yes (> 90%)*	*Yes (10–49%)*	No	No
(Surgical) masks	No	No	*Yes (> 90%)*	*Yes (< 10%)*	No	*Yes (10–49%)*
Laboratory (2017)						
Screening technique CPK	Culture/PCR, after broth enrichment	Culture/PCR, directly on clinical sample & Culture, after broth enrichment	Culture, directly from clinical sample	Culture, directly from clinical sample	Culture, directly from clinical sample & phenotypic confirmatory test	Culture, directly from clinical sample
Starting molecular typing of CPK	N = 2	*Always*	N > 2	* N.A.*	*In case of clinical or epidemiological need (cluster/ outbreak)*	N > 2
Molecular typing method of CPK	MLVA	Molecular typing is outsourced	RAPD, NGS	N.A.	Molecular typing is outsourced (RAPD, NGS/WGS, MLST)	NGS/WGS, MLST
Cleaning and disinfection						
Replacing separation curtains after discharge	Yes	*No*	Yes	Yes	*N.A.*	*No*
Disposables in the isolation room are discarded after discharge	Yes	Yes	Yes	Yes	Yes	Yes

Remarkable differences between de hospitals are depicted in *italic.* * Mentioned percentage is the self-reported adherence to own IPC policy. ^§^ Taking preventive cultures of persons with increased risk of HRMO, because they have been in contact with a confirmed positive case. ^¥^ Definition primary/index case: The first indicated patient in whom a clinical or screening sample was unexpectedly positive for a certain HRMO. Definition secondary case: A patient with the same HRMO as the primary/index case and is linked in time and place to the primary/index case. Definition outbreak: Two or more similar HRMO cases linked in time and place. Abbreviations; ECDC: European Centre for Disease Prevention and Control. CAESAR: WHO Central Asian and Eastern European Surveillance of Antimicrobial Resistance. IPC: infection prevention and control. HRMO: Highly resistant microorganisms. CPK: Carbapenemase-producing *Klebsiella pneumoniae*. N.D.: No data. N.A: Not applicable. PCR: Polymerase chain reaction. MLVA: Multiple-locus variable number tandem-repeat analysis. LAMP: Loop-mediated isothermal amplification. RAPD: Random amplified polymorphic DNA. NGS: Next generation sequencing. WGS: Whole genome sequencing. MLST: Multi-locus sequence typing. EMC: Erasmus MC University Medical Centre in Rotterdam, The Netherlands. KSK: Kardinal Schwarzenberg Klinikum in Salzburg, Austria. VGS: Vienna General Hospital in Vienna, Austria. ERU: Erciyes University in Kayseri, Turkey. INMI: National Institute for Infectious Diseases ‘Lazzaro Spallanzani’ in Rome, Italy. TGH: Tzaneio General Hospital in Piraeus, Greece

From the hospitals (EMC, KSK, VGS) in low prevalence countries, only the EMC always performed targeted screening (Table 2a). KSK always performed molecular typing of CPK and labelled patients in their electronic health record, but did not isolate these patients. Furthermore, VGS used more PPE than EMC and KSK.

Concerning the hospitals in high prevalence countries, ERU always performed targeted screening and used more PPE than INMI and TGH (Table 2a). However, ERU did not perform molecular typing of CPK. Other remarkable differences between the hospitals are depicted in italic font in Table 2a.

### CPPA specific IPC measures

The Netherlands was indicated by the ECDC as lowest prevalence country for CPPA. However, VGS and KSK reported a lower median self-reported prevalence of CPPA than the EMC (Table 2b, Additional file 4). VGS, KSK and INMI were situated in countries classified by the ECDC as medium prevalence countries. ERU and TGH reported the highest median prevalence and were also situated in high prevalence countries.

**Table 2:** **b.** Comparing national IPC policies on CPPA

	EMC (Netherlands)	VGS (Austria)	KSK (Austria)	INMI (Italy)	ERU (Turkey)	TGH (Greece)
**Prevalence according to ECDC maps 2015 and CAESAR 2016 (13, 14)**	3.7%	12.2%	12.2%	23.0%	32%	40%
	Low	Medium	Medium	Medium	High	High
**Median self-reported prevalence of HRMO per hospital per year, regardless of sample site (range 2014–2016)**	20 (13–29)	5 (0–17)	13 (0–16)	23 (17–31)	467 (378–557)	52 (43–61)
Targeted screening^§^						
Primary case^¥^ - targeted screening on hospitalized patients	Yes (> 90%)*	Yes (> 90%)	Yes (> 90%)	Yes (50–90%)	*Yes (> 90%)*	*No*
Primary case - targeted screening on discharged patients	*Yes (> 90%)*	No	No	No	*Yes (50–90%)*	*No*
Secondary case – targeted screening on hospitalized patients	*Yes (> 90%)*	Yes (> 90%)	Yes (> 90%)	Yes (> 90%)	*Yes (50–90%)*	*No*
Secondary case – targeted screening on discharged patients	*Yes (> 90%)*	No	No	No	*Yes (50–90%)*	*No*
Outbreak – targeted screening on hospitalized patients	*Yes (> 90%)*	Yes (> 90%)	Yes (> 90%)	Yes (> 90%)	*Yes (50–90%)*	*No*
Outbreak – targeted screening on discharged patients	*Yes (> 90%)*	No	No	*Yes (50–90%)*	*Yes (50–90%)*	*No*
Labelling						
Isolation label for CPPA-positive patients	Yes (> 90%)	Yes (50–90%)	*Yes, but without HRMO specification (50–90%)*	Yes (> 90%)	Yes (> 90%)	Yes (50–90%)
Number of negative cultures before lifting label	6, during one year	*Upon discharge (but stays archived digitally)*	N.D.	3	3, one week apart	2
Isolation measures						
Isolation in multi-bedroom with blocking of the beds	No	Yes (50–90%)	Yes (> 90%)	Yes (> 90%)	Yes (50–90%)	*No*
Isolation in single bedroom without anteroom	Yes (> 90%)	Yes (> 90%)	No	No	Yes (50–90%)	*No*
Isolation in single bedroom with anteroom	No	No	No	Yes (> 90%)	Yes (10–49%)	*No*
Personal protective equipment						
Non-sterile gloves	Yes (> 90%)	*Yes (> 90%)*	Yes (> 90%)	Yes (> 90%)	*Yes (> 90%)*	Yes (50–90%)
Disposable gowns	Yes (> 90%)	*Yes (> 90%)*	Yes (> 90%)	Yes (> 90%)	*Yes (> 90%)*	No
Caps	No	*Yes (> 90%)*	No	No	*Yes (10–49%)*	No
(Surgical) masks	No	*Yes (> 90%)*	No	No	*Yes (< 10%)*	No
Laboratory (2017)						
Screening technique CPPA	Culture/PCR, after broth enrichment	Culture, directly from clinical sample	Culture/PCR, directly on clinical sample & culture, after broth enrichment	Culture, directly from clinical sample	*N.A.*	Culture, directly from clinical sample
Starting molecular typing of CPPA	N = 2	N > 2	N > 2	*In case of clinical or epidemiological need (cluster/ outbreak)*	*N.A.*	*N.A.*
Molecular typing method of CPPA	MLVA	N.A.	Molecular typing is outsourced	Molecular typing is outsourced (RAPD, NGS/WGS, MLST)	N.A.	N.A.
Cleaning and disinfection						
Replacing separation curtains after discharge	Yes	Yes	No	N.A.	Yes	No
Disposables in the isolation room are discarded after discharge	Yes	Yes	*No*	Yes	Yes	Yes

Remarkable differences between de hospitals are depicted in *italic.* * Mentioned percentage is the self-reported adherence to own IPC policy. ^§^ Taking preventive cultures of persons with increased risk of HRMO, because they have been in contact with a confirmed positive case. ^¥^ Definition primary/index case: The first indicated patient in whom a clinical or screening sample was unexpectedly positive for a certain HRMO. Definition secondary case: A patient with the same HRMO as the primary/index case and is linked in time and place to the primary/index case. Definition outbreak: Two or more similar HRMO cases linked in time and place. Abbreviations; ECDC: European Centre for Disease Prevention and Control. CAESAR: WHO Central Asian and Eastern European Surveillance of Antimicrobial Resistance. IPC: infection prevention and control. HRMO: Highly resistant microorganisms. CPPA: Carbapenemase-producing *Pseudomonas aeruginosa.* N.D.: No data. N.A: Not applicable. PCR: Polymerase chain reaction. MLVA: Multiple-locus variable number tandem-repeat analysis. LAMP: Loop-mediated isothermal amplification. RAPD: Random amplified polymorphic DNA. NGS: Next generation sequencing. WGS: Whole genome sequencing. MLST: Multi-locus sequence typing. EMC: Erasmus MC University Medical Centre in Rotterdam, The Netherlands. VGS: Vienna General Hospital in Vienna, Austria. KSK: Kardinal Schwarzenberg Klinikum in Salzburg, Austria. INMI: National Institute for Infectious Diseases ‘Lazzaro Spallanzani’ in Rome, Italy. ERU: Erciyes University in Kayseri, Turkey. TGH: Tzaneio General Hospital in Piraeus, Greece

From the hospitals in low (EMC) and medium prevalence countries (VGS, KSK, INMI), defined by the ECDC, only EMC always performed targeted screening (Table 2b). Furthermore, VGS lifted the isolation label of a patient upon discharge and used more PPE than the other three hospitals.

Concerning ERU and TGH, hospitals in high prevalence countries, ERU always performed targeted screening and used more PPE than TGH. TGH did not perform targeted screening and did not isolate CPPA positive patients. Both hospitals did not perform molecular typing of CPPA (Table 2b). Other remarkable differences between the hospitals are depicted in italic font in Table 2b.

### VRE specific IPC measures

The EMC, KSK and VGS were according to the ECDC situated in low prevalence countries, while INMI, ERU and TGH were situated in medium prevalence countries (Table 2c, Additional file S4). However, KSK, INMI and EMC actually reported the lowest median prevalence of VRE, while TGH, ERU and VGS reported the highest median prevalence of VRE (Table 2c, Additional file 4).

**Table 2:** **c.** Comparing national IPC policies on VRE

	EMC (Netherlands)	KSK (Austria)	VGS (Austria)	INMI (Italy)	ERU (Turkey)	TGH (Greece)
**Prevalence according to ECDC maps 2015 and CAESAR 2016 (13, 14)**	1.9%	3.1%	3.1%	11.2%	16%	20%
	Low	Low	Low	Medium	Medium	Medium
**Median self-reported prevalence of HRMO per hospital per year, regardless of sample site (range 2014–2016)**	33 (9–47)	0 (0–0)	48 (48–114)	11 (3–23)	48 (42–51)	38 (31–47)
Targeted screening^§^						
Primary case^¥^ - targeted screening on hospitalized patients	Yes (> 90%)*	Yes (> 90%)	Yes (> 90%)	*No*	*Yes (> 90%)*	*No*
Primary case - targeted screening on discharged patients	*Yes (> 90%)*	No	No	No	*Yes (50–90%)*	No
Secondary case – targeted screening on hospitalized patients	*Yes (> 90%)*	Yes (> 90%)	Yes (> 90%)	Yes (> 90%)	*Yes (50–90%)*	*No*
Secondary case – targeted screening on discharged patients	*Yes (> 90%)*	No	No	No	*Yes (50–90%)*	No
Outbreak – targeted screening on hospitalized patients	*Yes (> 90%)*	Yes (> 90%)	Yes (> 90%)	Yes (> 90%)	*Yes (50–90%)*	Yes (50–90%)
Outbreak – targeted screening on discharged patients	*Yes (> 90%)*	No	No	*Yes (50–90%)*	*Yes (50–90%)*	No
Labelling						
Isolation label for VRE-positive patients	Yes (> 90%)	Yes, but without HRMO specification (50–90%)	Yes (> 90%)	Yes (> 90%)	Yes (> 90%)	Yes (> 90%)
Number of negative cultures before lifting label	6, during one year	N.D.	*Upon discharge (but stays archived digitally)*	2	3, one week apart	2
Isolation measures						
Isolation in multi-bedroom with blocking of the beds	No	*No*	Yes (50–90%)	Yes (> 90%)	Yes (50–90%)	*No*
Isolation in single bedroom without anteroom	Yes (> 90%)	*No*	Yes (> 90%)	No	Yes (50–90%)	*No*
Isolation in single bedroom with anteroom	No	*No*	No	Yes (> 90%)	Yes (10–49%)	*No*
Personal protective equipment						
Non-sterile gloves	Yes (> 90%)	Yes (> 90%)	Yes (> 90%)	Yes (> 90%)	*Yes (> 90%)*	Yes (50–90%)
Disposable gowns	Yes (> 90%)	Yes (> 90%)	*No*	Yes (> 90%)	*Yes (> 90%)*	Yes (50–90%)
Caps	No	No	Yes (> 90%)	No	*Yes (10–49%)*	No
(Surgical) masks	No	No	Yes (> 90%)	No	*Yes (< 10%)*	No
Laboratory (2017)						
Screening technique VRE	Culture/PCR, after broth enrichment. Suspension on vancomycin screenings agar 10 ul	Culture, directly from clinical sample & culture, after broth enrichment.	Culture/PCR, directly on clinical sample.	Culture, directly from clinical sample.	Culture, directly from clinical sample	Culture, directly from clinical sample.
Starting molecular typing of VRE	N = 2	N > 2	N > 2	In case of clinical or epidemiological need (cluster/ outbreak)	*N.A.*	*N.A.*
Molecular typing method of VRE	Molecular typing is outsourced	Molecular typing is outsourced	GeneXpert (Cepheid)	Molecular typing is outsourced (RAPD, NGS/WGS)	N.A.	N.A.
Cleaning and disinfection						
Replacing separation curtains after discharge	Yes	No	Yes	N.A.	Yes	No
Disposables in the isolation room are discarded after discharge	Yes	Yes	Yes	Yes	Yes	Yes

Remarkable differences between de hospitals are depicted in *italic.* * Mentioned percentage is the self-reported adherence to own IPC policy. ^§^ Taking preventive cultures of persons with increased risk of HRMO, because they have been in contact with a confirmed positive case. ^¥^ Definition primary/index case: The first indicated patient in whom a clinical or screening sample was unexpectedly positive for a certain HRMO. Definition secondary case: A patient with the same HRMO as the primary/index case and is linked in time and place to the primary/index case. Definition outbreak: Two or more similar HRMO cases linked in time and place. Abbreviations; ECDC: European Centre for Disease Prevention and Control. CAESAR: WHO Central Asian and Eastern European Surveillance of Antimicrobial Resistance. IPC: infection prevention and control. HRMO: Highly resistant microorganisms. VRE: vancomycin-resistant *Enterococcus faecium*. N.D.: No data. N.A: Not applicable. PCR: Polymerase chain reaction. MLVA: Multiple-locus variable number tandem-repeat analysis. LAMP: Loop-mediated isothermal amplification. RAPD: Random amplified polymorphic DNA. NGS: Next generation sequencing. WGS: Whole genome sequencing. MLST: Multi-locus sequence typing. EMC: Erasmus MC University Medical Centre in Rotterdam, The Netherlands. KSK: Kardinal Schwarzenberg Klinikum in Salzburg, Austria. VGS: Vienna General Hospital in Vienna, Austria. INMI: National Institute for Infectious Diseases ‘Lazzaro Spallanzani’ in Rome, Italy. ERU: Erciyes University in Kayseri, Turkey. TGH: Tzaneio General Hospital in Piraeus, Greece.

Of the three hospitals in low prevalence countries, only EMC always performed targeted screening (Table 2c). Furthermore, KSK did not isolate VRE-positive patients, in contrast to EMC and VGS.

From the hospitals in medium prevalence countries, ERU always performed targeted screening and used more PPE than INMI and TGH (Table 2c). TGH did not isolate VRE-positive patients. Furthermore, both ERU and TGH did not perform molecular typing. Other remarkable differences between the hospitals are depicted in italic font in Table 2c.

### International IPC guidelines

All three international guidelines recommend risk-based screening, but differ in the circumstances to do so (Additional file 2). Furthermore, only the CDC provides the recommendation to targetly screen hospitalized patients, after identifying a primary or secondary case, which is in line with the IPC policy of most hospitals. None of the three international guidelines provide recommendations on targeted screening of discharged patients. The recommended number of negative cultures before lifting the isolation label, differs per international guideline but also per HRMO. This difference is also seen in the IPC policies of the hospitals. Overall, the international guidelines do provide general recommendations on CPK and CPPA, but recommendations on VRE are often lacking.

### Number of IPC measures per hospital

**Fig. 1:**
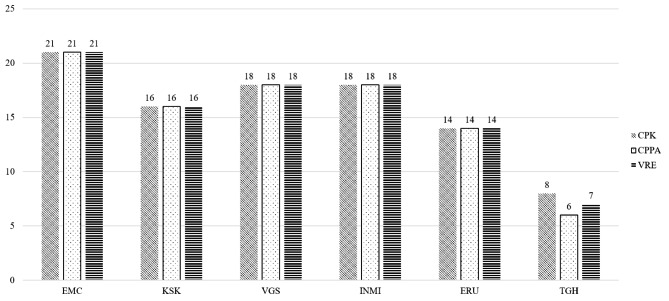
Number of implemented infection prevention and control measures in each hospital. Hospitals were only rewarded points when they reported an adherence of more than 90% to the IPC measure. Dichotomous yes/no questions: No = 0 points, Yes = 1 point. Abbreviations; CPK: Carbapenemase-producing *Klebsiella pneumoniae*, CPPA: Carbapenemase-producing *Pseudomonas aeruginosa*, VRE: vancomycin-resistant *Enterococcus faecium*, EMC: Erasmus MC University Medical Center in Rotterdam, The Netherlands. KSK: Kardinal Schwarzenberg Klinikum in Salzburg, Austria. VGS: Vienna General Hospital in Vienna, Austria. INMI: National Institute for Infectious Diseases ‘Lazzaro Spallanzani’ in Rome, Italy. ERU: Erciyes University in Kayseri, Turkey. TGH: Tzaneio General Hospital in Piraeus, Greece.

EMC implemented 21 IPC measures per HRMO (Fig. 1, Additional file 5). KSK implemented a total of 16 IPC measures, while VGS (located in the same country) implemented 18 IPC measures per HRMO (Fig. 1, Additional file 5). INMI also implemented 18 IPC measures, but was located in a country with medium/high prevalence. ERU and TGH were also located in countries with medium/high prevalence and implemented 14 (ERU) and 7 (median of TGH) IPC measures per HRMO, whereby TGH did not implement the same number of IPC measures for every HRMO (Fig. 1, Additional file 5).

Hospitals in low prevalence countries (i.e. as categorised by the ECDC) more often reported an adherence of > 90% (Tables 2a, 2b, 2c). This in contrast to hospitals in countries with medium or high prevalence, which more often showed adherence rates of 10–49% or 50–90%.

## Discussion

This study showed that for CPK and CPPA, the hospitals with the highest self-reported prevalence, implemented the least IPC measures in their hospitals. There was no clear relation between the self-reported prevalence of VRE and the number of implemented IPC measures. Furthermore, the hospitals with the lowest self-reported prevalence often reported the highest adherence to their own IPC policies.

The EMC implemented the most IPC measures (i.e. 21) in their hospital, but was not the hospital with the lowest median self-reported prevalence for any of the HRMO. KSK, VGS and INMI reported lower prevalence rates for the three HRMO, while implementing fewer IPC measures (i.e. KSK 16, VGS 18, INMI 18). This difference might be explained by the fact that KSK and INMI are a clinic and a national institute, while VGS and EMC are a general hospital and university medical centre. Willemsen et al. (2011) found that the incidence density of patients with HRMO was higher in university hospitals, since university hospitals often provide more specialized care and perform more complex care [7]. Furthermore, ERU reported the highest median prevalence for all three HRMO, but did not implement the least IPC measures. However, since TGH did not perform targeted screening for CPPA and VRE, the reported median prevalence of TGH could have been an underestimation and could in reality have been higher. This is in line with Vuichard-Gysin et al. (2022), who showed an increase of 57% in VRE detection due to an increase in admission screening [25].

This study also showed that there were differences in the HRMO IPC policies of the different hospitals. For example, KSK performed targeted screening, microbial typing and labelling of patients with CPK and VRE, but did not isolate these patients, in contrast to, for example EMC and VGS. However, considering that KSK indicated that CPK and VRE only started to emerge in their hospital in 2017, it is likely that KSK implemented fewer IPC measures for these HRMO than hospitals where these HRMO were already endemic. Differences in IPC policy between hospitals could also be explained by hospital organisation, bed occupancy, and type of hospital [4, 7]. It might also be helpful if international guidelines provide more uniform and detailed IPC recommendations. We found that the international guidelines sometimes gave different or even no recommendations on certain IPC measures (e.g. lifting an isolation label or targeted screening when identifying a primary or secondary case or during an outbreak).

Finally, this study showed that hospitals with higher self-reported prevalence of HRMO, often less adhered to their IPC measures. ERU (Turkey) and TGH (Greece) reported in general a high prevalence of HRMO and a low adherence to IPC measures. This is in line with the study of Tacconelli et al. (2019), who found that hospitals in Southern Europe often reported a low adherence to isolation measures, with as main reason budget restrictions [6]. The low adherence of ERU and TGH with IPC measures might therefore be explained by the fact that they do not have the financial resources to adhere to or implement more IPC measures. TGH for example labelled CPPA and VRE-positive patients, but did not isolate them nor performed molecular typing.

### Strengths and limitations

First, in addition to asking the hospitals about their IPC policy, we also asked the hospitals’ adherence to their own policy. In this way we collected accurate information about the implemented IPC measures for each HRMO. Furthermore, we included hospitals in such a way that countries with a low, medium, and high prevalence were represented in the study. Since the hospitals also provided prevalence data themselves over the last three years, we could use reliable first-hand data. Third, we focussed on HRMO indicated as critical (CPK and CPPA) and high (VRE) according to the WHO priority pathogens list [26]. Another strength is that despite the survey being extensive and detailed, the survey was completed by all the participants.

A first limitation is that the number of included hospitals is too small to demonstrate an association between a hospital’s IPC policy and their HRMO prevalence. Furthermore, we did not correct for type of hospital or gross domestic product per country, nor did we ask the hospitals about the local price of IPC measures. It could have been the case that hospitals with a high prevalence and few IPC measures wanted to implement more measures, but did not have the financial or organisational resources. The third limitation is that all data is self-reported and that it was filled out by only one person in each hospital. This may have led to bias, because hospitals could have given socially desirable answers. However, since this bias applied to all hospitals, we think that the effects on our results are negligible. Lastly, we took not into account the effects of standard precautions (e.g. hand hygiene compliance, personal protective equipment etc.) on the HRMO prevalence. Although standard precautions probably would have had an impact on the HRMO prevalence, it was beyond the scope of this study and not feasible to retrospectively collect the data.

## Conclusion

With the exception of VRE, the hospitals with the highest self-reported prevalence of the included HRMO, implemented in general the least IPC measures. The hospitals with the lowest HRMO prevalence, implemented the most IPC measures and also had a higher adherence to their own IPC policy. This study showed that in general, hospitals make different choices in their IPC policy, which could be due to the endemicity of specific HRMO or the lack of logistic or financial resources of a hospital. Furthermore, it could be helpful to invest in achieving a high adherence to the implemented IPC policy, as this could result in a reduction of the HRMO prevalence.

## Electronic supplementary material

Below is the link to the electronic supplementary material.

Supplementary Material 1

## Data Availability

The datasets used and/or analysed during the current study are available from the corresponding author on reasonable request.

## List of abbreviations

IPC: Infection prevention and control
HRMO: Highly resistant microorganisms
CPK: Carbapenemase-producing *Klebsiella pneumoniae*
CPPA: Carbapenemase-producing *Pseudomonas aeruginosa*
VRE: Vancomycin-resistant *Enterococcus faecium*
WHO: World Health Organisation
CDC: Centres for Disease Control and prevention
ECDC: European Centre for Disease Prevention and Control
ESCMID: European Society of Clinical Microbiology and Infectious Diseases
PPE: Personal protective equipment
EMC: Erasmus MC University Medical Centre in Rotterdam, The Netherlands
ERU: Erciyes University in Kayseri, Turkey
KSK: Kardinal Schwarzenberg Klinikum in Salzburg, Austria
INMI: National Institute for Infectious Diseases ‘Lazzaro Spallanzani’ in Rome, Italy
TGH: Tzaneio General Hospital in Piraeus, Greece
VGS: Vienna General Hospital in Vienna, Austria
CAESAR: Central Asian and Eastern European Surveillance of Antimicrobial Resistance
ESGNI: ESCMID Study Group for Nosocomial Infections
