# Correction: Infection prevention and control policies in hospitals and prevalence of highly resistant microorganisms: an international comparative study

**DOI:** 10.1186/s13756-023-01221-3

**Published:** 2023-03-29

**Authors:** Manon D. van Dijk, Anne F. Voor in ’t holt, Emine Alp, Markus Hell, Nicola Petrosillo, Elisabeth Presterl, Athanasios Tsakris, Juliëtte A. Severin, Margreet C. Vos, ESCMID Study Group for Nosocomial Infections (ESGNI)

**Affiliations:** 1 Department of Medical Microbiology and Infectious Diseases, Erasmus MC University Medical Centre Rotterdam, P.O. Box 2040, 3000 CA Rotterdam, The Netherlands; 2 Department of Infectious Diseases and Clinical Microbiology, Medical Faculty, Erciyes University, Kayseri, Turkey; 3 Department of Infectious Diseases and Clinical Microbiology, Medical Faculty, Ankara Yıldırım Beyazıt University, Ankara, Turkey; 4 Department of Clinical Microbiology and Infection Control, MEDILAB-Academic Teaching Laboratories, Paracelsus Medical University, Salzburg, Austria; 5 Teaching Hospital, Kardinal Schwarzenberg Klinikum, Paracelsus Medical University, Schwarzach, Austria; 6 Clinical and Research Department for Infectious Diseases, National Institute for Infectious Diseases “Lazzaro Spallanzani”, Rome, Italy; 7 Department of Infection Control, University Hospital Campus Bio-Medico, Rome, Italy; 8 Department of Infection Control and Hospital Epidemiology, Medical University of Vienna, Vienna, Austria; 9 Department of Microbiology, Medical School, University of Athens, Athens, Greece; 10 ESCMID Study Group for Nosocomial Infections (ESGNI), ESCMID Study Group for Nosocomial Infections (ESGNI), Basel, Switzerland

**Correction: Antimicrobial Resistance & Infection Control (2022) 11:152** 10.1186/s13756-022-01165-0

Following publication of the original article, the ESCMID Study Group for Nosocomial Infections (ESGNI) was mistakenly added as a separate author, whereas all authors were acting on behalf of the study group. The author list has been updated in this correction article and the original article [1] has been corrected. Additionally, the article contained numerous minor typos which have since been amended.
