# Supplementary material for: Infection prevention and control policies in hospitals and prevalence of highly resistant microorganisms: an international comparative study
Source: Antimicrob Resist Infect Control. 2022 Dec 6;11:152. doi: 10.1186/s13756-022-01165-0 (PMC9727845; doi:10.1186/s13756-022-01165-0)
Supplement: Supplementary file 1 — Supplementary Material 1 [file 13756_2022_1165_MOESM1_ESM.docx]

**Index additional files**

[Additional file 1: The online survey 2](#_Toc102477702)

[Additional file 2: Comparison between the IPC policy of six European hospitals and international IPC guidelines 87](#_Toc102477703)

[Additional file 3: Definitions primary and secondary case and an outbreak 95](#_Toc102477704)

[Additional file 4: Overview of the prevalence of HRMO per hospital per year 98](#_Toc102477705)

[Additional file 5: Implemented infection prevention and control measures per hospital 99](#_Toc102477706)

#### Additional file 1: The online survey

**Costs and effects of prevention policies for highly resistant microorganisms**

**Project: 'PRICE-HRMO'** Costs and effects of **PR**event**I**on poli**C**i**E**s for **H**ighly **R**esistant **M**icro**O**rganisms

*Important to remember:*

*1. All questions should be answered in English*

*2. If there are any uncertainties, please consult the 'Explanatory document' or contact us via e-mail (*[*price.hrmo@erasmusmc.nl*](mailto:price.hrmo@erasmusmc.nl)*).*

Dear reader,

On behalf of the ESCMID Study Group of Nosocomial Infections (ESGNI), we would like to thank you for the interest and participation in our PRICE-HRMO project.The results of this study, will be used to gain insight in the costs and effects of prevention policies of highly resistant microorganisms (HRMO).

At the end of this questionnaire you are asked to fill in your name and email address. We ask this to be able to contact you about the questionnaire, to send a financial compensation and to inform you about the results of the research. Furthermore, this link is an invitation on hospital level and we ask you to not forward this questionnaire to other institutions.

 Important notes about the questionnaire:

- Answers can be modified at any time
- It is possible to pause and save the questionnaire, simply by clicking on 'resume later' at the bottom of the questionnaire. You have to give your name and email address and think of a password. When you want to proceed, just click on the link in the email, enter your name and password and you can continue where you left off.
- You can return to previous questions by clicking on the 'previous' button.

If you have any questions or suggestions for this study, please contact us at [price.hrmo@erasmusmc.nl](mailto:price.hrmo@erasmusmc.nl)

Yours sincerely,

Prof. dr. Margreet C. Vos and Drs. Manon D. van Dijk

Department of Medical Microbiology and Infectious Diseases

Erasmus University Medical Center Rotterdam

*There are 269 questions in this survey*

**General questions**

In this section we would like you to answer general questions about your hospital and provide us with contact information.

N.B.: When you do not have the information, please fill in 999.

**Country:**

Please choose **only one** of the following:

[country options]

**City:**

[Please write your answer here]

**What is the complete name of your hospital?**

[Please write your answer here]

**Type of hospital**

Please choose **all** that apply:


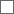
 Teaching hospital


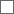
 General hospital


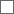
 Other, please specify::

**For which department do you work?**

[Please write your answer here]

**General questions about your hospital**

In this section we would like you to answer questions regarding the size of your hospital, the number of clinical hospital days and clinical hospital admissions for the years 2014, 2015 and 2016.

N.B.: When you do not have the information, please fill in 999.

**How many infection control practitioners has your hospital employed?**
[Please write your answer here] infection control practitioners

**How many infection control practitioners are on average present per day?**
[Please write your answer here] infection control practitioners

**How many doctors*, specialized in infectious diseases, has your hospital employed?**
[Please write your answer here] doctors

* Medical microbiologists and infectious disease specialists

**How many doctors*, specialized in infectious diseases, are on average present per day?** [Please write your answer here] doctors

* Medical microbiologists and infectious disease specialists

**How many beds are available for patient care in your hospital?**
[Please write your answer here] hospital beds

**What is the total number of intensive care beds in your hospital?**
[Please write your answer here] IC beds

* The total number of IC beds for adults, children and neonatals combined

**What is the number of single bedrooms in your hospital?**
[Please write your answer here] single bedrooms

* Single bedroom = a bedroom that is intended to accomodate a single bed and occupancy of one person

**What is the number of single bedrooms with anteroom in your hospital?**
[Please write your answer here] single bedrooms with anteroom

* Anteroom = room adjacent to the actual single bedroom, with or without pressure difference

**How many patients are on average in one patientroom?**
[Please write your answer here] patients

**Total number of clinical hospital days and admissions* (excluding day-care)**

|  | 2014 | 2015 | 2016 |
| --- | --- | --- | --- |
| Total number of clinical hospital days |  |  |  |
| Total number of clinical hospital admissions |  |  |  |

* Clinical hospital days/admissions = admission to a hospital, excluding one-day admissions / day-care

**The following questions will be repeated for 2014, 2015 and 2016**

Highly Resistant Microorganisms 2014

These questions refer to the period: 01/01/2014 - 31/12/2014 (day/month/year)

N.B.: When you do not have the information, please fill in 999.

**In 2014, did your microbial laboratory test for the presence of resistance genes (e.g. carbapenemase genes like OXA, NDM, KPC or VanA, VanB)?**

Please choose **only one** of the following:


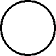
 No


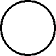
 Yes


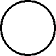
 No data/ not known

**Carbapenemase producing *Klebsiella pneumoniae* (CPK) in 2014**

Number of patients tested positive for CPK in 2014 (any culture)

[Please write your answer here]

Number of patients tested positive for CPK (any culture), 48 hours after hospitalization in 2014

[Please write your answer here]

Number of patients with a CPK positive blood culture in 2014

[Please write your answer here]

Number of patients with a CPK positive blood culture, 48 hours after hospitalization in 2014

[Please write your answer here]

Number of patients who were targetly screened for CPK carriage in 2014

[Please write your answer here]

Number of positive CPK cases that came from targeted screening in 2014

[Please write your answer here]

**Carbapenemase producing *Pseudomonas aeruginosa*(CPPA) in 2014**

Number of patients tested positive for CPPA in 2014 (any culture)

[Please write your answer here]

Number of patients tested positive for CPPA (any culture), 48 hours after hospitalization in 2014

[Please write your answer here]

Number of patients with a CPPA positive blood culture in 2014

[Please write your answer here]

Number of patients with a CPPA positive blood culture, 48 hours after hospitalization in 2014

[Please write your answer here]

Number of patients who were targetly screened for CPPA carriage in 2014

[Please write your answer here]

Number of positive CPPA cases that came from targeted screening in 2014

[Please write your answer here]

**Vancomycin resistant *Enterococcus faecium* (VRE, only VanA and/or VanB) in 2014** Number of patients tested positive for VRE in 2014 (any culture)

[Please write your answer here]

Number of patients tested positive for VRE (any culture), 48 hours after hospitalization in 2014

[Please write your answer here]

Number of patients with a VRE positive blood culture in 2014

[Please write your answer here]

Number of patients with a VRE positive blood culture, 48 hours after hospitalization in 2014

[Please write your answer here]

Number of patients who were targetly screened for VRE carriage in 2014

[Please write your answer here]

Number of positive VRE cases that came from targeted screening in 2014

[Please write your answer here]

Highly Resistant Microorganisms 2015

These questions refer to the period: 01/01/2015 - 31/12/2015 (day/month/year)

N.B.: When you do not have the information, please fill in 999.

**In 2015, did your microbial laboratory test for the presence of resistance genes (e.g. carbapenemase genes like OXA, NDM, KPC or VanA, VanB)?**

Please choose **only one** of the following:


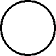
 No


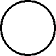
 Yes


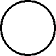
 No data/ not known

**Carbapenemase producing *Klebsiella pneumoniae* (CPK) in 2015**

Number of patients tested positive for CPK in 2015 (any culture)

[Please write your answer here]

Number of patients tested positive for CPK (any culture), 48 hours after hospitalization in 2015

[Please write your answer here]

Number of patients with a CPK positive blood culture in 2015

[Please write your answer here]

Number of patients with a CPK positive blood culture, 48 hours after hospitalization in 2015

[Please write your answer here]

Number of patients who were targetly screened for CPK carriage in 2015

[Please write your answer here]

Number of positive CPK cases that came from targeted screening in 2015

[Please write your answer here]

**Carbapenemase producing Pseudomonas aeruginosa (CPPA) in 2015**

Number of patients tested positive for CPPA in 2015 (any culture)

[Please write your answer here]

Number of patients tested positive for CPPA (any culture), 48 hours after hospitalization in 2015

[Please write your answer here]

Number of patients with a CPPA positive blood culture in 2015

[Please write your answer here]

Number of patients with a CPPA positive blood culture, 48 hours after hospitalization in 2015

[Please write your answer here]

Number of patients who were targetly screened for CPPA carriage in 2015

[Please write your answer here]

Number of positive CPPA cases that came from targeted screening in 2015

[Please write your answer here]

**Vancomycin resistant Enterococcus faecium (VRE, only VanA and/or VanB) in 2015**

Number of patients tested positive for VRE in 2015 (any culture)

[Please write your answer here]

Number of patients tested positive for VRE (any culture), 48 hours after hospitalization in 2015

[Please write your answer here]

Number of patients with a VRE positive blood culture in 2015

[Please write your answer here]

Number of patients with a VRE positive blood culture, 48 hours after hospitalization in 2015

[Please write your answer here]

Number of patients who were targetly screened for VRE carriage in 2015

[Please write your answer here]

Number of positive VRE cases that came from targeted screening in 2015

[Please write your answer here]

Highly Resistant Microorganisms 2016

These questions refer to the period: 01/01/2016 - 31/12/2016 (day/month/year)

N.B.: When you do not have the information, please fill in 999.

### In **2016**, did your microbial laboratory test for the presence of resistance genes (e.g. carbapenemase genes like OXA, NDM, KPC or VanA, VanB)?

Please choose **only one** of the following:


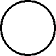
 No


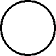
 Yes


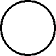
 No data/ not known

### Carbapenemase producing Klebsiella pneumoniae (CPK) in 2016

Number of patients tested positive for CPK in 2016 (any culture)

[Please write your answer here]

Number of patients tested positive for CPK (any culture), 48 hours after hospitalization in 2016

[Please write your answer here]

Number of patients with a CPK positive blood culture in 2016

[Please write your answer here]

Number of patients with a CPK positive blood culture, 48 hours after hospitalization in 2016

[Please write your answer here]

Number of patients who were targetly screened for CPK carriage in 2016

[Please write your answer here]

Number of positive CPK cases that came from targeted screening in 2016

[Please write your answer here]

**Carbapenemase producing Pseudomonas aeruginosa (CPPA) in 2016**

Number of patients tested positive for CPPA in 2016 (any culture)

[Please write your answer here]

Number of patients tested positive for CPPA (any culture), 48 hours after hospitalization in 2016

[Please write your answer here]

Number of patients with a CPPA positive blood culture in 2016

[Please write your answer here]

Number of patients with a CPPA positive blood culture, 48 hours after hospitalization in 2016

[Please write your answer here]

Number of patients who were targetly screened for CPPA carriage in 2016

[Please write your answer here]

Number of positive CPPA cases that came from targeted screening in 2016

[Please write your answer here]

**Vancomycin resistant Enterococcus faecium (VRE, only VanA and/or VanB) in 2016**

Number of patients tested positive for VRE in 2016 (any culture)

[Please write your answer here]

Number of patients tested positive for VRE (any culture), 48 hours after hospitalization in 2016

[Please write your answer here]

Number of patients with a VRE positive blood culture in 2016

[Please write your answer here]

Number of patients with a VRE positive blood culture, 48 hours after hospitalization in 2016

[Please write your answer here]

Number of patients who were targetly screened for VRE carriage in 2016

[Please write your answer here]

Number of positive VRE cases that came from targeted screening in 2016

[Please write your answer here]

## Highly Resistant Microorganisms (HRMO) 2017

Highly Resistant Microorganisms 2017

This section has only one question that determines what the following questions, about the prevention policy, will be about

When you check 'yes', the following questions will refer to:

   - Carbapenemase producing Klebsiella pneumoniae (CPK)

   - Carbapenemase producing Pseudomonas aeruginosa (CPPA)

   - Vancomycin resistant Enterococcus faecium (VRE, only VanA and/or VanB)

As determined by the presence of genes.

When you check 'no', all the following questions will refer to:

   - Carbapenem resistant Klebsiella pneumoniae (CRK)

   - Carbapenem resistant Pseudomonas aeruginosa (CRPA)

   - Vancomycin resistant Enterococcus faecium (VRE)

As determined by susceptibility testing.

### In **2017**, does your microbial laboratory test for the presence of resistance genes (e.g. carbapenemase genes like OXA, NDM, KPC or VanA, VanB)? *

Please choose **only one** of the following:


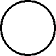
Yes


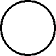
No

N.B. This is an important question that determines the rest of the questionnaire.

### When you test for resistance genes for (for example) only two out of three HRMO, please specify this here.

[Please write your answer here]

## Prevention policy - laboratory

In this section we would like to know which methods are currently used in your laboratory.

N.B.: When you do not have the information, please fill in 999.

### Which technique is currently used to screen for CPK?

Please choose **all** that apply:


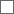
Culture, directly from clinical sample (tissue, fluid or swab)


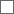
Culture, after broth enrichment


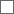
PCR, directly on clinical sample (tissue, fluid or swab)


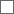
PCR, after broth enrichment


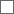
Other, please specify::

CPK = Carbapenemase producing Klebsiella pneumoniae

* More options possible

### What method is currently used for molecular typing of CPK?

Please choose **all** that apply:


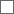
PFGE


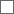
AFLP


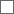
MLVA


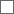
RAPD


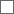
NGS / WGS


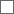
Molecular typing is outsourced


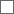
We do not perform molecular typing


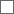
Other, please specify::

CPK = Carbapenemase producing Klebsiella pneumoniae

* More options possible

### Which technique is currently used to screen for CPPA?

Please choose **all** that apply:


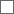
Culture, directly from clinical sample (tissue, fluid or swab)


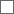
Culture, after broth enrichment


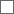
PCR, directly on clinical sample (tissue, fluid or swab)


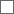
PCR, after broth enrichment


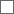
Other, please specify::

CPPA = Carbapenemase producing Pseudomonas aeruginosa

* More options possible

### What method is currently used for molecular typing of CPPA?

Please choose **all** that apply:


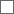
PFGE


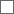
AFLP


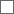
MLVA


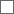
RAPD


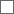
NGS / WGS


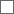
Molecular typing is outsourced


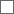
We do not perform molecular typing


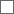
Other, please specify::

CPPA = Carbapenemase producing Pseudomonas aeruginosa

* More options possible

### Which technique is currently used to screen for VRE?

Please choose **all** that apply:


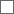
Culture, directly from clinical sample (tissue, fluid or swab)


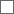
Culture, after broth enrichment


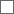
PCR, directly on clinical sample (tissue, fluid or swab)


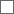
PCR, after broth enrichment


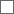
Other, please specify::

VRE = Vancomycin resistant Enterococcus faecium (only VanA and/or VanB)

* More options possible

### What method is currently used for molecular typing of VRE?

Please choose **all** that apply:


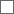
PFGE


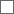
AFLP


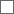
MLVA


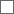
RAPD


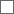
NGS / WGS


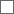
Molecular typing is outsourced


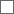
We do not perform molecular typing


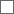
Other, please specify::

VRE = Vancomycin resistant Enterococcus faecium (only VanA and/or VanB)

* More options possible

## Prevention policy - risk groups

In this section we want to know what your current policy is regarding risk groups for HRMO. To be clear, we first want to know what you should do according to policy. Secondly, we want to know to what extent you actually comply with your policy.

N.B: When you do not have the information, please fill in 999.

### Do you triage a patient to identify if this patient has an increased risk of HRMO?

Please choose **all** that apply:


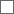
Yes, always upon entry


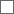
Yes, during hospitalization period


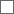
Only when there are indications that a patient could have an increased risk of HRMO


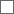
Never


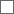
Other, please specify::

* Triage = the process of determining if a patient has an increased risk of being a HRMO carrier.

### When a patient has been hospitalized less than two months ago (for more than 24 hours) in a hospital abroad, do you targetly screen this patient for HRMO carriage when he/she is admitted to your hospital?

Please choose **only one** of the following:


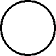
Yes


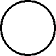
No

* Targeted screening = Taking preventive cultures of persons with increased risk of HRMO, because they have been in contact with a confirmed positive case or because they are a risk group for HRMO (e.g. hospitalized abroad, farmer etc.)

### Compliance rate of this policy

Please choose **only one** of the following:


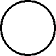
All of the time (>90%)


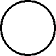
Usually (50-90%)


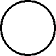
Sometimes (10-49%)


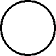
Rarely/never (<10%)


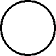
No data

### When a patient has been hospitalized in another hospital with a HRMO-outbreak (which is not yet under control), do you targetly screen this patient for HRMO carriage when he/she is admitted to your hospital?

Please choose **only one** of the following:


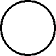
Yes


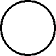
No

* Targeted screening = taking preventive cultures of persons with increased risk of HRMO, because they have been in contact with a confirmed positive case or because they are a risk group for HRMO (e.g. hospitalized abroad, farmer etc.)

### Compliance rate of this policy

Please choose **only one** of the following:


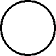
All of the time (>90%)


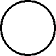
Usually (50-90%)


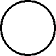
Sometimes (10-49%)


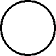
Rarely/never (<10%)


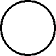
No data

### Are patients, at risk for HRMO, placed in pre-emptive isolation awaiting their screening results?

Please choose **only one** of the following:


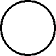
Yes


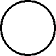
No

### Compliance rate of this policy

Please choose **only one** of the following:


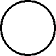
All of the time (>90%)


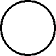
Usually (50-90%)


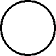
Sometimes (10-49%)


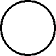
Rarely/never (<10%)


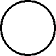
No data

## Prevention policy - Isolation period

In this section we want to know what your current policy is regarding the isolation period of a patient. To be clear, we first want to know what you should do according to policy. Secondly, we want to know to what extent you actually comply with your policy.

N.B.: When you do not have the information, please fill in 999.

### When do you place a patient in isolation?*

Please choose **all** that apply:


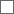
When the patient already has an isolation indication in its medical record (labeled)


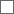
When the triage shows that the patient has an increased risk of HRMO


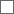
When cultures show that the patient is positive for a HRMO


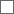
Patients are never placed in isolation


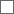
Other, please specify::

Triage = the process of determining if a patient has an increased risk of being a HRMO carrier.

* More options possible

### (Approximately the) total number of isolation days for:

|  | 2014 | 2015 | 2016 |
| --- | --- | --- | --- |
| Carbapenemase producing Klebsiella pneumoniae |  |  |  |
| Carbapenemase producing Pseudomonas aeruginosa |  |  |  |
| Vancomycin resistant Enterococcus faecium (only VanA and/or VanB) |  |  |  |

N.B.: When you do not have the information, please fill in 999.

### The patient is labeled with the specific HRMO in its medical record in case of:

Please choose **all** that apply:


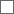
Carbapenemase producing Klebsiella pneumoniae


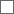
Carbapenemase producing Pseudomonas aeruginosa


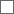
Vancomycin resistant Enterococcus faecium (only VanA and/or VanB)


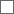
The patient is labeled with HRMO, but without specification for which HRMO


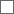
Not applicable

### Compliance rate of CPK labeling

Please choose **only one** of the following:


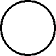
All of the time (>90%)


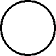
Usually (50-90%)


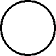
Sometimes (10-49%)


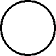
Rarely/never (<10%)


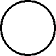
No data

### Compliance rate of CPPA labeling

Please choose **only one** of the following:


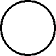
All of the time (>90%)


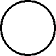
Usually (50-90%)


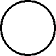
Sometimes (10-49%)


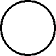
Rarely/never (<10%)


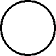
No data

### Compliance rate of VRE labeling

Please choose **only one** of the following:


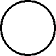
All of the time (>90%)

Usually (50-90%)

Sometimes (10-49%)

Rarely/never (<10%)

No data

### Compliance rate of unspecified labeling

Please choose **only one** of the following:

All of the time (>90%)

Usually (50-90%)

Sometimes (10-49%)

Rarely/never (<10%)

No data

**After how many negative cultures or after what time is the label, of the specific HRMO, removed from the medical record?**

**N.B.: Mention in your answer if you refer to 'negative cultures' or 'days/months/years'.**

Comment only when you choose an answer.

Please choose all that apply and provide a comment:

- Carbapenemase producing *Klebsiella pneumoniae*

[Please write your answer here]

- Carbapenemase producing *Pseudomonas aeruginosa*

[Please write your answer here]

- Vancomycin resistant *Enterococcus faecium* (only VanA and/or VanB)

[Please write your answer here]

## Prevention policy - Infection control precautions

In this section we want to know what your current policy is regarding infection control precautions. To be clear, we first want to know what you should do according to policy. Secondly, we want to know to what extent you actually comply with your policy.

N.B.: More options possible. When you do not have the information, please fill in 999.

### Patient is placed in isolation in a multi bedroom with blocking of the other beds in case of:

Please choose **all** that apply:

Carbapenemase producing Klebsiella pneumoniae

Carbapenemase producing Pseudomonas aeruginosa

Vancomycin resistant Enterococcus faecium(only VanA and/or VanB)

None of the above

### Compliance rate of this CPK isolation policy

Please choose **only one** of the following:

All of the time (>90%)

Usually (50-90%)

Sometimes (10-49%)

Rarely/never (<10%)

No data

### Compliance rate of this CPPA isolation policy

Please choose **only one** of the following:

All of the time (>90%)

Usually (50-90%)

Sometimes (10-49%)

Rarely/never (<10%)

No data

### Compliance rate of this VRE isolation policy

Please choose **only one** of the following:

All of the time (>90%)

Usually (50-90%)

Sometimes (10-49%)

Rarely/never (<10%)

No data

### Patient is placed in isolation in a single bedroom without anteroom* in case of:

Please choose **all** that apply:

Carbapenemase producing Klebsiella pneumoniae

Carbapenemase producing Pseudomonas aeruginosa

Vancomycin resistant Enterococcus faecium(only vanA and/or VanB)

None of the above

* Anteroom = room adjacent to the actual isolation room, with or without any pressure difference.

###

### Compliance rate of this CPK isolation policy

Please choose **only one** of the following:

All of the time (>90%)

Usually (50-90%)

Sometimes (10-49%)

Rarely/never (<10%)

No data

### Compliance rate of this CPPA isolation policy

Please choose **only one** of the following:

All of the time (>90%)

Usually (50-90%)

Sometimes (10-49%)

Rarely/never (<10%)

No data

### Compliance rate of this VRE isolation policy

Please choose **only one** of the following:

All of the time (>90%)

Usually (50-90%)

Sometimes (10-49%)

Rarely/never (<10%)

No data

### Patient is placed in isolation in a single bedroom with anteroom* in case of:

Please choose **all** that apply:

Carbapenemase producing Klebsiella pneumoniae

Carbapenemase producing Pseudomonas aeruginosa

Vancomycin resistant Enterococcus faecium (only VanA and/or VanB)

None of the above

* Anteroom = room adjacent to the actual isolation room, with or without any pressure difference

### Compliance rate of this CPK isolation policy

Please choose **only one** of the following:

All of the time (>90%)

Usually (50-90%)

Sometimes (10-49%)

Rarely/never (<10%)

No data

### Compliance rate of this CPPA isolation policy

Please choose **only one** of the following:

All of the time (>90%)

Usually (50-90%)

Sometimes (10-49%)

Rarely/never (<10%)

No data

### Compliance rate of this VRE isolation policy

Please choose **only one** of the following:

All of the time (>90%)

Usually (50-90%)

Sometimes (10-49%)

Rarely/never (<10%)

No data

### Non-sterile gloves are used for all patient contact* in case of:

Please choose **all** that apply:

Carbapenemase producing Klebsiella pneumoniae

Carbapenemase producing Pseudomonas aeruginosa

Vancomycin resistant Enterococcus faecium (only VanA and/or VanB)

None of the above

* All patient contact = entering or working in the same room as where the HRMO positive patient is hospitalized

###

### Compliance rate of this CPK glove policy

Please choose **only one** of the following:

All of the time (>90%)

Usually (50-90%)

Sometimes (10-49%)

Rarely/never (<10%)

No data

Compliance rate of this CPPA glove policy

Please choose **only one** of the following:

All of the time (>90%)

Usually (50-90%)

Sometimes (10-49%)

Rarely/never (<10%)

No data

**Compliance rate of this VRE glove policy**

Please choose **only one** of the following:

All of the time (>90%)

Usually (50-90%)

Sometimes (10-49%)

Rarely/never (<10%)

No data

### Disposable gowns are used for all patient contact* in case of:

**Only answer this question if the following conditions are met:**
Please choose **all** that apply:

Carbapenemase producing Klebsiella pneumoniae

Carbapenemase producing Pseudomonas aeruginosa

Vancomycin resistant Enterococcus faecium (only VanA and/or VanB)

None of the above

* All patient contact = entering or working in the same room as where the HRMO positive patient is hospitalized

**Compliance rate of this CPK gown policy**

Please choose **only one** of the following:

All of the time (>90%)

Usually (50-90%)

Sometimes (10-49%)

Rarely/never (<10%)

No data

### Compliance rate of this CPPA gown policy

Please choose **only one** of the following:

All of the time (>90%)

Usually (50-90%)

Sometimes (10-49%)

Rarely/never (<10%)

No data

### Compliance rate of this VRE gown policy

Please choose **only one** of the following:

All of the time (>90%)

Usually (50-90%)

Sometimes (10-49%)

Rarely/never (<10%)

No data

### Caps are used for all patient contact* in case of:

Please choose **all** that apply:

Carbapenemase producing Klebsiella pneumoniae

Carbapenemase producing Pseudomonas aeruginosa

Vancomycin resistant Enterococcus faecium(only VanA and/or VanB)

None of the above

* All patient contact = entering or working in the same room as where the HRMO positive patient is hospitalized

### Compliance rate of this CPK cap policy

Please choose **only one** of the following:

All of the time (>90%)

Usually (50-90%)

Sometimes (10-49%)

Rarely/never (<10%)

No data

### Compliance rate of this CPPA cap policy

Please choose **only one** of the following:

All of the time (>90%)

Usually (50-90%)

Sometimes (10-49%)

Rarely/never (<10%)

No data

### Compliance rate of this VRE cap policy

Please choose **only one** of the following:

All of the time (>90%)

Usually (50-90%)

Sometimes (10-49%)

Rarely/never (<10%)

No data

### (Surgical) masks are used for all patient contact* in case of:

Please choose **all** that apply:

Carbapenemase producing Klebsiella pneumoniae

Carbapenemase producing Pseudomonas aeruginosa

Vancomycin resistant Enterococcus faecium (only VanA and/or VanB)

None of the above

* All patient contact = entering or working in the same room as where the HRMO positive patient is hospitalized

### Compliance rate of this CPK mask policy

Please choose **only one** of the following:

All of the time (>90%)

Usually (50-90%)

Sometimes (10-49%)

Rarely/never (<10%)

No data

### Compliance rate of this CPPA mask policy

Please choose **only one** of the following:

All of the time (>90%)

Usually (50-90%)

Sometimes (10-49%)

Rarely/never (<10%)

No data

### Compliance rate of this VRE mask policy

Please choose **only one** of the following:

All of the time (>90%)

Usually (50-90%)

Sometimes (10-49%)

Rarely/never (<10%)

No data

### FFP1 masks are used for all patient contact* in case of:

Please choose **all** that apply:

Carbapenemase producing Klebsiella pneumoniae

Carbapenemase producing Pseudomonas aeruginosa

Vancomycin resistant Enterococcus faecium (only VanA and/or VanB)

None of the above

* All patient contact = entering or working in the same room as where the HRMO positive patient is hospitalized

### Compliance rate of this CPK FFP1 mask policy

Please choose **only one** of the following:

All of the time (>90%)

Usually (50-90%)

Sometimes (10-49%)

Rarely/never (<10%)

No data

### Compliance rate of this CPPA FFP1 mask policy

Please choose **only one** of the following:

All of the time (>90%)

Usually (50-90%)

Sometimes (10-49%)

Rarely/never (<10%)

No data

**Compliance rate of this VRE FFP1 mask policy**

Please choose **only one** of the following:

All of the time (>90%)

Usually (50-90%)

Sometimes (10-49%)

Rarely/never (<10%)

No data

**FFP2 masks are used for all patient contact* in case of:**

Please choose **all** that apply:

Carbapenemase producing Klebsiella pneumoniae

Carbapenemase producing Pseudomonas aeruginosa

Vancomycin resistant Enterococcus faecium (only VanA and/or VanB)

None of the above

* All patient contact = entering or working in the same room as where the HRMO positive patient is hospitalized

**Compliance rate of this CPK FFP2 mask policy**

Please choose **only one** of the following:

All of the time (>90%)

Usually (50-90%)

Sometimes (10-49%)

Rarely/never (<10%)

No data

**Compliance rate of this CPPA FFP2 mask policy**

Please choose **only one** of the following:

All of the time (>90%)

Usually (50-90%)

Sometimes (10-49%)

Rarely/never (<10%)

No data

**Compliance rate of this VRE FFP2 mask policy**

Please choose **only one** of the following:

All of the time (>90%)

Usually (50-90%)

Sometimes (10-49%)

Rarely/never (<10%)

No data

## Prevention policy - Transmission

In this section we want to know what your current policy is regarding transmission of HRMO. To be clear, we first want to know what you should do according to policy. Secondly, we want to know to what extent you actually comply with your policy.

N.B.: When you do not have the information, please fill in 999.

**In your hospital, what is the definition of a primary case?**

**- Describe for clinical and screening sample**

**- Describe the epidemiological characteristics**

**- Describe if and which molecular typing is used**

[Please write your answer here]

**When a primary case is detected, targeted screening can be one of the measures you will take. In case of which HRMO do you perform targeted screening?**

**N.B. Check the HRMO if your hospital performs targeted screening after finding this specific HRMO in a primary case.**

Please choose **all** that apply:

Carbapenemase producing Klebsiella pneumoniae

Carbapenemase producing Pseudomonas aeruginosa

Vancomycin resistant Enterococcus faecium (only VanA and/or VanB)

None of the above

* Targeted screening = taking preventive cultures of persons with increased risk of HRMO, because they have been in contact with a confirmed positive case.

### Compliance rate of this CPK screening policy

Please choose **only one** of the following:

All of the time (>90%)

Usually (50-90%)

Sometimes (10-49%)

Rarely/never (<10%)

No data

### Compliance rate of this CPPA screening policy

Please choose **only one** of the following:

All of the time (>90%)

Usually (50-90%)

Sometimes (10-49%)

Rarely/never (<10%)

No data

### Compliance rate of this VRE screening policy

Please choose **only one** of the following:

All of the time (>90%)

Usually (50-90%)

Sometimes (10-49%)

Rarely/never (<10%)

No data

**After detecting a primary case, for which HRMO do you perform targeted screening on patients who are still hospitalized?**

**N.B. Check the HRMO if your hospital performs targeted screening (on hospitalized patients) after finding this specific HRMO in a primary case.**

Please choose **all** that apply:

Carbapenemase producing Klebsiella pneumoniae

Carbapenemase producing Pseudomonas aeruginosa

Vancomycin resistant Enterococcus faecium (only VanA and/or VanB)

None of the above

* Targeted screening = taking preventive cultures of persons with increased risk of HRMO, because they have been in contact with a confirmed positive case.

### General compliance rate of this screening policy of hospitalized patients

Please choose **only one** of the following:

All of the time (>90%)

Usually (50-90%)

Sometimes (10-49%)

Rarely/never (<10%)

No data

**After detecting a primary case, for which HRMO do you perform targeted screening on patients who are already discharged?**

**N.B. Check the HRMO if your hospital performs targeted screening (on discharged patients) after finding this specific HRMO in a primary case.**

Please choose **all** that apply:

Carbapenemase producing Klebsiella pneumoniae

Carbapenemase producing Pseudomonas aeruginosa

Vancomycin resistant Enterococcus faecium (only VanA and/or VanB)

None of the above

* Discharged: 1. Relocated to another hospital, 2. Went home.

* Targeted screening = taking preventive cultures of persons with increased risk of HRMO, because they have been in contact with a confirmed positive case.

### General compliance rate of this screening policy of discharged patients

Please choose **only one** of the following:

All of the time (>90%)

Usually (50-90%)

Sometimes (10-49%)

Rarely/never (<10%)

No data

**In your hospital, what is the definition of a secondary case?**

**- Describe for clinical and screening sample**

**- Describe the epidemiological characteristics**

**- Describe if and which molecular typing is used**

[Please write your answer here]

**When a secondary case is detected, targeted screening can be one of the measures you will take. In case of which HRMO do you perform targeted screening?**

**N.B. Check the HRMO if your hospital performs targeted screening after finding this specific HRMO in a secondary case.**

Please choose **all** that apply:

Carbapenemase producing Klebsiella pneumoniae

Carbapenemase producing Pseudomonas aeruginosa

Vancomycin resistant Enterococcus faecium (only VanA and/or VanB)

None of the above

* Targeted screening = taking preventive cultures of persons with increased risk of HRMO, because they have been in contact with a confirmed positive case.

### Compliance rate of this CPK screening policy

Please choose **only one** of the following:

All of the time (>90%)

Usually (50-90%)

Sometimes (10-49%)

Rarely/never (<10%)

No data

### Compliance rate of this CPPA screening policy

Please choose **only one** of the following:

All of the time (>90%)

Usually (50-90%)

Sometimes (10-49%)

Rarely/never (<10%)

No data

### Compliance rate of this VRE screening policy

Please choose **only one** of the following:

All of the time (>90%)

Usually (50-90%)

Sometimes (10-49%)

Rarely/never (<10%)

No data

**After detecting a secondary case, for which HRMO do you perform targeted screening on patients who are still hospitalized?**

**N.B. Check the HRMO if your hospital performs targeted screening (on hospitalized patients) after finding this specific HRMO in a secondary case.**

Please choose **all** that apply:

- Carbapenemase producing Klebsiella pneumoniae
- Carbapenemase producing Pseudomonas aeruginosa
- Vancomycin resistant Enterococcus faecium (only VanA and/or VanB)
- None of the above

* Targeted screening = taking preventive cultures of persons with increased risk of HRMO, because they have been in contact with a confirmed positive case.

### General compliance rate of this screening policy of hospitalized patients

Please choose **only one** of the following:

All of the time (>90%)

Usually (50-90%)

Sometimes (10-49%)

Rarely/never (<10%)

No data

**After detecting a secondary case, for which HRMO do you perform targeted screening on patients who are already discharged?**

**N.B. Check the HRMO if your hospital performs targeted screening (on discharged patients) after finding this specific HRMO in a secondary case.**

Please choose **all** that apply:

Carbapenemase producing Klebsiella pneumoniae

Carbapenemase producing Pseudomonas aeruginosa

Vancomycin resistant Enterococcus faecium (only VanA and/or VanB)

None of the above

* Discharged: 1. Relocated to another hospital, 2. Went home.

* Targeted screening = taking preventive cultures of persons with increased risk of HRMO, because they have been in contact with a confirmed positive case.

### General compliance rate of this screening policy of discharged patients

**Only answer this question if the following conditions are met:**

Please choose **only one** of the following:

All of the time (>90%)

Usually (50-90%)

Sometimes (10-49%)

Rarely/never (<10%)

No data

**In your hospital, what is the definition of an outbreak?**

**- Describe for clinical and screening sample**

**- Describe the epidemiological characteristics**

**- Describe if and which molecular typing is used**

**Make a distinction between CPK, CPPA an VRE (only VanA and/or VanB)**

[Please write your answer here]

**When more than two linked positive cases are detected, targeted screening can be one of the measures you will take. In case of which HRMO do you perform targeted screening?**

**N.B. Check the HRMO if your hospital performs targeted screening after finding this specific HRMO in more than two linked positive cases.**

Please choose **all** that apply:

Carbapenemase producing Klebsiella pneumoniae

Carbapenemase producing Pseudomonas aeruginosa

Vancomycin resistant Enterococcus faecium (only VanA and/or VanB)

None of the above

* Targeted screening = taking preventive cultures of persons with increased risk of HRMO, because they have been in contact with a confirmed positive case.

* Linked positive cases = linked in time and place and/or strains are similar according to the molecular typing procedure of the center involved.

**Compliance rate of this CPK screening policy**

Please choose **only one** of the following:

All of the time (>90%)

Usually (50-90%)

Sometimes (10-49%)

Rarely/never (<10%)

No data

**Compliance of this CPPA screening policy**

Please choose **only one** of the following:

All of the time (>90%)

Usually (50-90%)

Sometimes (10-49%)

Rarely/never (<10%)

No data

**Compliance rate of this VRE screening policy**

Please choose **only one** of the following:

All of the time (>90%)

Usually (50-90%)

Sometimes (10-49%)

Rarely/never (<10%)

No data

**After detecting more than two linked positive cases, for which HRMO do you perform targeted screening on patients who are still hospitalized?**

**N.B. Check the HRMO if your hospital performs targeted screening (on hospitalized patients) after finding this specific HRMO in more than two linked positive cases.**

Please choose **all** that apply:

Carbapenemase producing Klebsiella pneumoniae

Carbapenemase producing Pseudomonas aeruginosa

Vancomycin resistant Enterococcus faecium (only VanA and/or VanB)

None of the above

* Targeted screening = taking preventive cultures of persons with increased risk of HRMO, because they have been in contact with a confirmed positive case.

* Linked positive cases = linked in time and place and/or strains are similar according to the molecular typing procedure of the center involved.

**General compliance rate of this screening policy of hospitalized patients**

Please choose **only one** of the following:

All of the time (>90%)

Usually (50-90%)

Sometimes (10-49%)

Rarely/never (<10%)

No data

**After detecting more than two linked positive cases, for which HRMO do you perform targeted screening on patients who are already discharged ?**

**N.B. Check the HRMO if your hospital performs targeted screening (on discharged patients) after finding this specific HRMO in more than two linked positive cases.**

Please choose **all** that apply:

Carbapenemase producing Klebsiella pneumoniae

Carbapenemase producing Pseudomonas aeruginosa

Vancomycin resistant Enterococcus faecium (only VanA and/or VanB)

None of the above

* Discharged = 1. Relocated to another hospital, 2. Went home.

* Targeted screening = taking preventive cultures of persons with increased risk of HRMO, because they have been in contact with a confirmed positive case.

* Linked positive cases = linked in time and place and/or strains are similar according to the molecular typing procedure of the center involved.

**General compliance rate of this screening policy of discharged patients**

Please choose **only one** of the following:

All of the time (>90%)

Usually (50-90%)

Sometimes (10-49%)

Rarely/never (<10%)

No data

**Is extra staff employed during an outbreak?**

Please choose **only one** of the following:

No

Yes

No data

Other, please specify:

**When do you targetly screen healtcare workers?**

Please choose **all** that apply:

Healthcare workers are targetly screened when there are indications that they could have an increased risk of HRMO

Healthcare workers are targetly screened when a primary case is found

Healthcare workers are targetly screened when a secondary case is found

Healthcare workers are targetly screened during an outbreak

Healthcare workers are never targetly screened

It depends on the microorganisms whether healthcare workers are targetly screened

* Targeted screening = taking preventive cultures of persons with increased risk of HRMO, because they have been in contact with a confirmed positive case.

**How many linked positive cases do you accept before starting targeted screening?**

Carbapenemase producing Klebsiella pneumoniae

[Please write your answer here]

Carbapenemase producing Pseudomonas aeruginosa

[Please write your answer here]

Vancomycin resistant Enterococcus faecium (only VanA and/or VanB)

[Please write your answer here]

**When do you perform molecular typing?**

Please choose the appropriate response for each item:

|  | Always | N>2 | Never | Other (Please specify in the next question) |
| --- | --- | --- | --- | --- |
| CPK |  |  |  |  |
| CPPA |  |  |  |  |
| VRE (only VanA and/or VanB) |  |  |  |  |

CPK = Carbapenemase producing *Klebsiella pneumoniae*

CPPA = Carbapenemase producing *Pseudomonas aeruginosa*

VRE = Vancomycin resistant *Enterococcus faecium* (only VanA and/or VanB)

N = the number of linked positive cases

**When do you perform molecular typing, please specify your answer:**

[Please write your answer here]

## Prevention policy - cleaning

In this section we want to know what your policy is regarding cleaning of the isolation room. Check the answer that fits the most with what you actually do in your hospital.

N.B.: When you do not have the information, please fill in 999.

**Cleaning and disinfection of the hospital is outsourced**

Please choose **only one** of the following:

No

Yes

Other, please specify:

**During hospitalization, the isolation room is cleaned and/or disinfected*:**

Please choose **all** that apply:

cleaned dry, without a microfibre cloth

cleaned dry, with a microfibre cloth

cleaned wet, with a detergent

disinfected with chlorine/bleach

disinfected with quarternary ammonia

disinfected with hydrogen peroxide

disinfected with alcohol

none of the above

Other, please specify::

*More options possible

### During hospitalization, frequency of cleaning and/or disinfecting of the isolation room:

Please choose the appropriate response for each item:

|  | Once a week | Two times a week | Three times a week | Four times a week | Five times a week | Six times a week | Every day of the week | Only after discharge of the patient | Never |
| --- | --- | --- | --- | --- | --- | --- | --- | --- | --- |
| CPK |  |  |  |  |  |  |  |  |  |
| CPPA |  |  |  |  |  |  |  |  |  |
| VRE (only VanA and/or VanB) |  |  |  |  |  |  |  |  |  |

CPK = Carbapenemas producing Klebsiella pneumoniae

CPPA = Carbapenemase producing Pseudomonas aeruginosa

VRE = Vancomycin resistant Enterococcus faecium (only VanA and/or VanB)

Check if it applies to your policy for the specific HRMO.

### Disposables present in the isolation room, that can not be cleaned and/or disinfected, are thrown away after discharge of the patient in case of:

Please choose **all** that apply:

Carbapenemase producing Klebsiella pneumoniae

Carbapenemase producing Pseudomonas aeruginosa

Vancomycin resistant Enterococcus faecium (only VanA and/or VanB)

None of the above

Other, please specify::

Check if it applies to your policy for the specific HRMO.

###

### When present in the isolation room, separation curtains are replaced after discharge of the patient in case of:

Please choose **all** that apply:

Carbapenemase producing Klebsiella pneumoniae

Carbapenemase producing Pseudomonas aeruginosa

Vancomycin resistant Enterococcus faecium (only VanA and/or VanB)

None of the above

Other, please specify::

**After discharge, the isolation room is cleaned and/or disinfected*:**

Please choose **all** that apply:

cleaned dry, without a microfibre cloth

cleaned dry, with a microfibre cloth

cleaned wet, with a detergent

disinfected with chlorine/bleach

disinfected with quarternary ammonia

disinfected with hydrogen peroxide

disinfected with alcohol

none of the above

Other, please specify::

*More options possible

**Prevention policy - policy changes**

In this section we want to know if your HRMO prevention policy changed during 2014, 2015 or 2016. If your policy has been adjusted over these years, we would like to know what the biggest changes were.

Has your HRMO prevention policy been changed in 2014? Please choose **all** that apply:

Carbapenemase producing Klebsiella pneumoniae

Carbapenemase producing Pseudomonas aeruginosa

Vancomycin resistant Enterococcus faecium (only VanA and/or VanB)

No HRMO prevention policy was changed in 2014

### In 2014, what was changed in the CPK prevention policy?

[Please write your answer here]

### In 2014, what was changed in the CPPA prevention policy?

[Please write your answer here]

### In 2014, what was changed in the VRE prevention policy?

[Please write your answer here]

### Has your HRMO prevention policy been changed in 2015?

Please choose **all** that apply:

Carbapenemase producing Klebsiella pneumoniae

Carbapenemase producing Pseudomonas aeruginosa

Vancomycin resistant Enterococcus faecium (only VanA and/or VanB)

No HRMO prevention policy was changed in 2015

###

### In 2015, what was changed in the CPK prevention policy?

[Please write your answer here]

### In 2015, what was changed in the CPPA prevention policy?

[Please write your answer here]

### In 2015, what was changed in the VRE prevention policy?

[Please write your answer here]

### Has your HRMO prevention policy been changed in 2016?

Please choose **all** that apply:

Carbapenemase producing Klebsiella pneumoniae

Carbapenemase producing Pseudomonas aeruginosa

Vancomycin resistant Enterococcus faecium (only VanA and/or VanB)

No HRMO prevention policy was changed in 2016

### In 2016, what was changed in the CPK prevention policy?

[Please write your answer here]

### In 2016, what was changed in the CPPA prevention policy?

[Please write your answer here]

### In 2016, what was changed in the VRE prevention policy?

[Please write your answer here]

## *Thank you for filling in this questionnaire!*

When you are sure you completely filled in the questionnaire and you uploaded your own prevention policies* you can submit the questionnaire.

* preferably in English but when only available in your own language, please upload these files.

### Name:

[Please write your answer here]

* [Titulature] [First name(s)] [Surname(s)]

### E-mail address:

[Please write your answer here]

### Please upload your prevention policy (preferably in English) for: Carbapenemase producing Klebsiella pneumoniae

Please upload at most one file

Kindly attach the aforementioned documents along with the survey

### Please upload your prevention policy (preferably in English) for: Carbapenemase producing Pseudomonas aeruginosa

Please upload at most one file
Kindly attach the aforementioned documents along with the survey

### Please upload your prevention policy (preferably in English) for: Vancomycin resistant Enterococcus faecium

Please upload at most one file
Kindly attach the aforementioned documents along with the survey

#### Additional file 2: Comparison between the IPC policy of six European hospitals and international IPC guidelines

| *Triage* | *ESCMID* | *WHO* | *CDC* | *EMC (Netherlands)* | *KSK (Austria)* | *VGS (Austria)* | *INMI (Italy)* | *ERU (Turkey)* | *TGH (Greece)* |
| --- | --- | --- | --- | --- | --- | --- | --- | --- | --- |
| Triage of patients | Yes, but only high-risk patients in endemic/ outbreak setting | Yes, during community outbreaks of communicable diseases | No, only active surveillance of risk factors among patients upon admission | Yes, always upon entry. | Only with high-risk patients | Yes, upon entry and during hospitalization period | Yes, always upon entry. | Yes, upon entry and during hospitalization period | Only with high-risk patients |
| *Targeted screening* | *ESCMID* | *WHO* | *CDC* | *EMC (Netherlands)* | *KSK (Austria)* | *VGS (Austria)* | *INMI (Italy)* | *ERU (Turkey)* | *TGH (Greece)* |
| Actively screen patients that were hospitalized <2 months ago for >24h in hospital abroad. | N.D. | Yes | Only for carbapenem-resistant *Enterobacteriaceae* | Yes (50-90%)* | Yes (>90%) | Yes (<10%) | Yes (50-90%) | Yes (50-90%) | Yes (50-90%) |
| Actively screen patients that were hospitalized in hospitals with HRMO-outbreak. | N.D. | Yes | Yes | Yes (50-90%) | Yes (>90%) | Yes (>90%) | Yes (>90%) | Yes (50-90%) | Yes (>90%) |
| Place high-risk patients in pre-emptive isolation awaiting their screening results | Yes | Yes | Only for carbapenem-resistant *Enterobacteriaceae* | Yes (>90%) | Yes (>90%) | Yes (>90%) | Yes (>90%) | No | Yes (50-90%) |
| Primary case - targeted screening on hospitalized patients | N.D. | N.D. | Only for carbapenem-resistant *Enterobacteriaceae* | Yes (>90%) | Yes (>90%) | Yes (>90%) | Only for CPK and CPPA (50-90%) | Yes (>90%) | Only for CPK (10-49%) |
| Primary case - targeted screening on discharged patients | N.D. | N.D. | N.D. | Yes (>90%) | No | No | No | Yes  (50-90%) | No |
| Secondary case – targeted screening on hospitalized patients | N.D. | N.D. | Only for carbapenem-resistant *Enterobacteriaceae* | Yes (>90%) | Yes (>90%) | Yes (>90%) | Yes (>90%) | Yes  (50-90%) | Only for CPK (50-90%) |
| Secondary case – targeted screening on discharged patients | N.D. | N.D. | N.D. | Yes (>90%) | No | No | No | Yes  (50-90%) | No |
| Outbreak – targeted screening on hospitalized patients | CPK/ CPPA: Yes, VRE: N.D. | Yes | CPK: Yes, CPPA/ VRE: N.D. | Yes (>90%) | Yes (>90%) | Yes (>90%) | Yes (>90%) | Yes  (50-90%) | CPK/ VRE: Yes (50-90%), CPPA: No. |
| Outbreak – targeted screening on discharged patients | N.D. | N.D. | N.D. | Yes (>90%) | No | No | Yes  (50-90%) | Yes  (50-90%) | No |
| Screening healthcare workers | During an outbreak | N.D. | HCW are not screened for carbapenem-resistant *Enterobacteriaceae* | N.A. | N.A. | N.A. | Depends on the HRMO | N.A. | During an outbreak |
| *Labelling* | *ESCMID* | *WHO* | *CDC* | *EMC (Netherlands)* | *KSK (Austria)* | *VGS (Austria)* | *INMI (Italy)* | *ERU (Turkey)* | *TGH (Greece)* |
| Isolation label for HRMO-positive patients | CPK/ CPPA: Yes, VRE: N.D. | CPK/ CPPA: Yes, VRE: N.D. | CPK/CPPA: Yes, VRE: N.D. | Yes (>90%) | Yes, but without HRMO specification (50-90%) | Yes, CPK/ CPPA (50-90%), VRE (>90%) | Yes (>90%) | Yes (>90%) | Yes, CPK/ VRE (>90%), CPPA (50-90%) |
| Number of negative cultures before lifting label | ≥3, during 2 weeks | CPK/ CPPA: 2. VRE: N.D. | CPK: max. 12 months. CPPA/ VRE: N.D. | 6, during 1 year | N.D. | Upon discharge (but stays archived digitally) | CPK/ CPPA: 3. VRE: 2 | 3, apart from 1 week | CPK: 3, CPPA/ VRE: 2 |
| *Isolation measures* | *ESCMID* | *WHO* | *CDC* | *EMC (Netherlands)* | *KSK (Austria)* | *VGS (Austria)* | *INMI (Italy)* | *ERU (Turkey)* | *TGH (Greece)* |
| Isolation of patients who already have an isolation label in their patient record | Yes | N.D. | N.D. | Yes | Yes | Yes | Yes | Yes | No |
| Isolation of patient when triage shows that the patient has increased risk of HRMO | N.D. | Yes | Yes | Yes | Yes | No | Yes | Yes | No |
| Isolation of patient with positive HRMO culture | Yes | N.D. | Yes | Yes | Yes | Yes | Yes | Yes | Yes |
| Isolation in multi-bedroom with blocking of the beds | CPK/ CPPA: No. VRE: N.D. | No | No | No | CPK/ VRE: No. CPPA: Yes (>90%) | Yes  (50-90%) | Yes (>90%) | Yes  (50-90%) | No |
| Isolation in single bedroom without anteroom | CPK/ CPPA: Yes. VRE: N.D. | Yes | Yes | Yes (>90%) | No | Yes (>90%) | No | Yes  (50-90%) | CPK: Yes (50-90%). CPPA/ VRE: No. |
| Isolation in single bedroom with anteroom | CPK/ CPPA: No. VRE: N.D. | No | No | No | No | No | Yes (>90%) | Yes  (10-49%) | No |
| *Personal protective equipment* | *ESCMID* | *WHO* | *CDC* | *EMC (Netherlands)* | *KSK (Austria)* | *VGS (Austria)* | *INMI (Italy)* | *ERU (Turkey)* | *TGH (Greece)* |
| Non-sterile gloves | Yes | Yes | Yes | Yes (>90%) | Yes (>90%) | Yes (>90%) | Yes (>90%) | Yes (>90%) | Yes  (50-90%) |
| Disposable gowns | Yes | Yes | Yes | Yes (>90%) | Yes (>90%) | CPK/ CPPA: Yes (>90%). VRE: No. | Yes (>90%) | Yes (>90%) | CPK/ VRE: Yes (50-90%). CPPA: No. |
| Caps | CPK/ CPPA: No. VRE: N.D | No | No | No | No | Yes (>90%) | No | Yes (10-49%) | No |
| (Surgical) masks | CPK/ CPPA: No. VRE: N.D | No | No | No | No | Yes (>90%) | No | Yes (<10%) | CPK: Yes (10-49%), CPPA/VRE: No. |
| *Laboratory (2017)* | *ESCMID* | *WHO* | *CDC* | *EMC (Netherlands)* | *KSK (Austria)* | *VGS (Austria)* | *INMI (Italy)* | *ERU (Turkey)* | *TGH (Greece)* |
| Screening technique CPK | Culture-based methodologies. | N.D. | No consensus on the optimal method. | Culture/PCR, after broth enrichment | Culture/PCR, directly on clinical sample. Culture, after broth enrichment | Culture, directly from clinical sample | Culture, directly from clinical sample, Phenotypic confirmatory test | Culture, directly from clinical sample | Culture, directly from clinical sample |
| Starting molecular typing of CPK | N.D. | N=2 | N.D. | N=2 | Always | N>2 | In case of clinical or epidemiological need (cluster/ outbreak) | N.A. | N>2 |
| Molecular typing method of CPK | N.D. | N.D. | PCR, MHT, Carba NP, metallo-β-lactamase testing | MLVA | Molecular typing is outsourced | RAPD, NGS | Molecular typing is outsourced (RAPD, NGS/WGS, MLST) | N.A. | NGS/WGS, MLST |
| Screening technique CPPA | Culture-based methodologies. | N.D. | No consensus on the optimal method. | Culture/PCR, after broth enrichment | Culture/PCR, directly on clinical sample. Culture, after broth enrichment | Culture, directly from clinical sample | Culture, directly from clinical sample | N.A. | Culture, directly from clinical sample |
| Starting molecular typing of CPPA | N.D. | N=2 | N.D. | N=2 | N>2 | N>2 | In case of clinical or epidemiological need (cluster/ outbreak) | N.A. | N.A. |
| Molecular typing method of CPPA | N.D. | N.D. | PCR, MHT, Carba NP, metallo-β-lactamase testing | MLVA | Molecular typing is outsourced | N.A. | Molecular typing is outsourced (RAPD, NGS/WGS, MLST) | N.A. | N.A. |
| Screening technique VRE | N.D. | N.D. | N.D. | Culture/PCR, after broth enrichment. Suspension on vancomycin screenings agar 10 ul | Culture, directly from clinical sample. Culture, after broth enrichment. | Culture/PCR, directly on clinical sample. | Culture, directly from clinical sample. | Culture, directly from clinical sample | Culture, directly from clinical sample. |
| Starting molecular typing of VRE | N.D. | N=2 | N.D. | N=2 | N>2 | N>2 | In case of clinical or epidemiological need (cluster/ outbreak) | N.A. | N.A. |
| Molecular typing method of VRE | N.D. | N.D. | N.D. | Molecular typing is outsourced | Molecular typing is outsourced | GeneXpert (Cepheid) | Molecular typing is outsourced (RAPD, NGS/WGS) | N.A. | N.A. |
| *Cleaning and disinfection* | *ESCMID* | *WHO* | *CDC* | *EMC (Netherlands)* | *KSK (Austria)* | *VGS (Austria)* | *INMI (Italy)* | *ERU (Turkey)* | *TGH (Greece)* |
| Cleaning isolation room, during hospitalization | Detergents or disinfectants | Cleaning with a detergent/disinfectant solution | N.D. | Dry, with microfibre cloth. Disinfected with chlorine/ bleach. Disinfected with alcohol. | Wet, with detergent. Disinfected with quarternary ammonia. | Dry, without microfibre cloth. Wet, with detergent. Disinfected with hydrogen peroxide. Small surfaces disinfected with alcohol. | Dry, with microfibre cloth. Disinfected with chlorine/bleach. | Disinfected with chlorine/bleach | Wet, with detergent. Disinfected with chlorine/bleach. Disinfected with alcohol |
| Cleaning isolation room, after discharge | Detergents or disinfectants. | Cleaning with a detergent/disinfectant solution | N.D. | Dry, with microfibre cloth. Disinfected with chlorine /bleach. Disinfected with alcohol | Disinfected with quarternary ammonia | Wet, with detergent. Disinfected with hydrogen peroxide. Disinfected with alcohol | Dry, with microfibre cloth. Disinfected with chlorine/bleach | Disinfected with chlorine/bleach | Wet, with detergent. Disinfected with chlorine/bleach. Disinfected with alcohol |
| Frequency of cleaning | N.D. | Daily | Daily | Daily | Daily | Daily | Daily | Daily | Daily |
| Replacing separation curtains after discharge | N.D. | Yes | N.D. | Yes | No | Curtains are cleaned and disinfected | N.A. | Yes | No |
| Disposables in the isolation room are discarded after discharge | Yes | N.D. | N.D. | Yes | CPK/ VRE: Yes. CPPA: No. | Yes | Yes | Yes | Yes |

* Mentioned percentage is the self-reported compliance with own IPC policy, 1. No data, 2. Rarely / never (<10%), 3.Sometimes (10-49%), 4. Usually (50-90%), and 5. All of the time (> 90% ). Abbreviations; IPC: infection prevention and control. HRMO: Highly resistant microorganisms. EMC: Erasmus MC University Medical Centre in Rotterdam, The Netherlands. KSK: Kardinal Schwarzenberg Klinikum in Salzburg, Austria. VGS: Vienna General Hospital in Vienna, Austria. INMI: National Institute for Infectious Diseaes ‘Lazzaro Spallanzani’ in Rome, Italy. ERU: Erciyes University in Kayseri, Turkey. TGH: Tzaneio General Hospital in Piraeus, Greece. CPK: carbapenemase-producing *Klebsiella pneumonia*, CPPA: carbapenemase-producing *Pseudomonas aeruginosa*, VRE: vancomycin-resistant *Enterococcus faecium*.

#### Additional file 3: Definitions primary and secondary case and an outbreak

**Definition primary case**

ESCMID: No data

WHO: No data

CDC: No data

EMC: ‘When for clinical as well as for a screening sample an unexpected HRMO positive culture is found it is our index patient. Targed screening is indicated. Roommates will be cultured and isolated. Patient hospitalized in the same department are cultured. No typing is indicated yet.’

INMI: ‘The person who first brings a disease into a group of people (cluster or outbreak); for many outbreaks, the primary case will never be known. The index case is the patient in an outbreak who is first diagnosed/noticed.’

TGH: ‘Patient screened positive for HRMO carriage upon admission or acquired HRMO closely not related to the circulating (in our hospital) strains during hospitalization. Screening sample: rectal swabMolecular typing: MLST (not performed regularly).’

VGS: ‘The primary (index) case is the initial patient that indicates the existence of a possible outbreak. Primary patients can be detected either via clinical parameters (e.g. disease progression or clinical symptoms indicative of that disease), or via targeted screening, or purely coincidental within the framework of clinical microbiological sampling. Primary cases are usually identified (labeled) as such retrospectively when a transmission to a secondary case has been established and a real outbreak has been detected. Molecular typing is performed via DiversiLab™ System (bioMérieux), an automated DNA fingerprinting system based on the rep-PCR electrophoresis technique’.

KSK: No data

ERU: No data

**Definition secondary case**

ESCMID: No data

WHO: No data

CDC: No data

EMC: ‘If there is a second positive HRMO with a link in time and place compared with the primary case. Molecular tying will be done.’

INMI;‘A patient with the same HRMO of an index (primary) case epidemiologically and/or biomolecularly linked’

TGH; ‘Patient screened negative for HRMO carriage upon admission and acquired HRMO closely related to the circulating (in our hospital) strains during hospitalization. Screening sample: rectal swabMolecular typing: MLST (not performed regularly).’

VGS; ‘The secondary case is the second laboratory-confirmed case with the disease in question during the outbreak. Clinical manifestation of the disease is not an absolute requirement for a case to be considered a secondary case. Secondary cases can manifest as silent carriers without clinical symptoms (e.g. VRE carriers) but high potential for disease transmission. Molecular typing is performed via DiversiLab™ System (bioMérieux), an automated DNA fingerprinting system based on the rep-PCR electrophoresis technique’.

KSK: No data

ERU: No data

**Definition outbreak**

ESCMID: ‘An unusual or unexpected increase of cases of infections due to MDR-GNB already isolated in the hospital or the emergence of cases of infection due to a new MDR-GNB, with or without molecular analysis of strains.’

WHO: ‘The occurrence of two or more similar cases relating to place and time is identified as a cluster or an outbreak and needs investigation to discover the route of transmission of infection, and possible sources of infection in order to apply measures to prevent further spread.’

CDC: No data

EMC: ‘If there is a transmission of an identical HRMO (n=2 or more) in a department with an epidemiological link, and the source is probably the department itself.’

INMI: ‘More than one secondary case’.

TGH: ‘Two or more linked positive cases harbouring closely related organisms clustered by time’.

VGS: ‘An outbreak is defined as such when at least two or more cases of a disease (or HRMO in clinical samples) in a particular place (hospital ward, outpatient clinic) are identified within a certain time frame (usually days, sometimes weeks). Outbreaks can comprise infectious diseases, HRMO transmission, or environmental sources. When an outbreak is suspected, other current patients in that place are screened specifically for the microorganism in question. An outbreak team is formed (at least 2 people) and communication with the ward or clinical setting in question is established immediately. CPK and CPPA outbreaks have not yet occurred at our hospital. Known carriers of either HRMO are immediately placed in isolation and prevention measures are taken. In the case of laboratory-confirmed VRE transmission, other patients will be subjected to targeted screening to identify other possible cases. Molecular typing is performed via DiversiLab™ System (bioMérieux), an automated DNA fingerprinting system based on the rep-PCR electrophoresis technique’.

KSK: ‘2 and more patients with same type of HRMO in the external lab they microarray chip techniques’.

ERU: No data

#### Additional file 4: Overview of the prevalence of HRMO per hospital per year

|  | |  |  | **EMC (Netherlands)** | | | **KSK (Austria)** | | | | **VGS (Austria)** | | | | **INMI (Italy)** | | | | **ERU (Turkey)** | | | | **TGH (Greece)** | | | |
| --- | --- | --- | --- | --- | --- | --- | --- | --- | --- | --- | --- | --- | --- | --- | --- | --- | --- | --- | --- | --- | --- | --- | --- | --- | --- | --- |
|  |  | |  | N total positive | N (%) positive after 48h | N positive after 48h per 10,000 admissions | | N total positive | N (%) positive after 48h | N positive after 48h per 10,000 admissions | | N total positive | N (%) positive after 48h | N positive after 48h per 10,000 admissions | | N total positive | N (%) positive after 48h | N positive after 48h per 10,000 admissions | | N total positive | N (%) positive after 48h | N positive after 48h per 10,000 admissions | | N Total positive | N (%) positive after 48h | N positive after 48h per 10,000 admissions |
| **CPK** | **2014** | | Any culture | 4 | 2 (50%) | 0,54 | | 0 | 0 | 0,00 | | 2 | 0 (0%) | 0,00 | | 69 | N.D. | N.D. | | 190 | 33 (17%) | 2,02 | | 156 | 133 (85%) | 63,93 |
|  |  | | *Blood culture* | *0* | *0* | *0,00* | | *0* | *0* | *0,00* | | *0* | *0* | *0,00* | | *5* | *5 (100%)* | *16,46* | | *71* | *9 (13%)* | *0,55* | | *40* | *38 (95%)* | *18,27* |
|  | **2015** | | Any culture | 2 | 1 (50%) | 0,27 | | 0 | 0 | 0,00 | | 3 | 2 (67%) | 0,19 | | 66 | 19 (29%) | 65,38 | | 382 | 80 (21%) | 4,71 | | 191 | 153 (80%) | 75,57 |
|  |  | | *Blood culture* | *0* | *0* | *0,00* | | *0* | *0* | *0,00* | | *0* | *0* | *0,00* | | *7* | *4 (57%)* | *13,76* | | *173* | *24 (14%)* | *1,41* | | *46* | *42 (91%)* | *20,74* |
|  | **2016** | | Any culture | 5 | 2 (40%) | 0,53 | | 0 | 0 | 0,00 | | 17 | 7 (41%) | 0,61 | | 34 | 10 (29%) | 32,36 | | 399 | 64 (16%) | 3,69 | | 178 | 150 (84%) | 73,22 |
|  |  | | *Blood culture* | *0* | *0* | *0,00* | | *0* | *0.* | *0,00* | | *N.D.* | *N.D.* | *N.D.* | | *1* | *0 (0%)* | *0,00* | | *123* | *18 (15%)* | *1,04* | | *32* | *27 (84%)* | *13,18* |
| **CPPA** | **2014** | | Any culture | 29 | 14 (48%) | 3,79 | | 0 | 0 | 0,00 | | 17 | 4 (24%) | 0,38 | | 31 | N.D. | N.D. | | 557 | 76 (14%) | 4,64 | | 43 | 37 (86%) | 17,79 |
|  |  | | *Blood culture* | *4* | *4 (100%)* | *1,08* | | *0* | *0.* | *0,00* | | *0* | *0 .* | *0,00* | | *0* | *0* | *0,00* | | *172* | *21 (12%)* | *1,28* | | *14* | *9 (64%)* | *4,33* |
|  | **2015** | | Any culture | 20 | 4 (20%) | 1,09 | | 13 | 13 (100%) | 4,38 | | 0 | 0 | 0,00 | | 17 | 3 (18%) | 10,32 | | 467 | 77 (16%) | 4,54 | | 61 | 49 (80%) | 24,20 |
|  |  | | *Blood culture* | *1* | *1 (100%)* | *0,27* | | *0* | *0* | *0,00* | | *0* | *0* | *0,00* | | *4* | *4 (100%)* | *13,76* | | *149* | *22 (15%)* | *1,30* | | *16* | *14 (88%)* | *6,91* |
|  | **2016** | | Any culture | 13 | 5 (38%) | 1,32 | | 16 | 16 (100%) | 5,45 | | 5 | 3 (60%) | 0,26 | | 23 | 9 (39%) | 29,13 | | 378 | 60 (16%) | 3,46 | | 52 | 44 (85%) | 21,48 |
|  |  | | *Blood culture* | *0* | *0* | *0,00* | | *0* | *0.* | *0,00* | | *N.D.* | *N.D.* | *N.D.* | | *2* | *1 (50%)* | *3,24* | | *68* | *15 (22%)* | *0,86* | | *10* | *10 (100%)* | *4,88* |
| **VRE** | **2014** | | Any culture | 9 | 3 (33%) | 0,81 | | 0 | 0 | 0,00 | | 48 | 10 (21%) | 0,94 | | 11 | N.D. | N.D. | | 42 | 10 (24%) | 0,61 | | 38 | 32 (84%) | 15,38 |
|  |  | | *Blood culture* | *2* | *1 (50%)* | *0,27* | | *0* | *0* | *0,00* | | *9* | *N.D.* | *N.D.* | | *1* | *0 (0%)* | *0,00* | | *26* | *7 (27%)* | *0,43* | | *4* | *3 (75%)* | *1,44* |
|  | **2015** | | Any culture | 47 | 18 (38%) | 4,89 | | 0 | 0 | 0,00 | | 48 | 7 (15%) | 0,66 | | 3 | 1 (33%) | 3,44 | | 48 | 9 (19%) | 0,53 | | 47 | 38 (81%) | 18,77 |
|  |  | | *Blood culture* | *2* | *2 (100%)* | *0,54* | | *0* | *0* | *0,00* | | *0* | *0* | *0,00* | | *0* | *0* | *0,00* | | *20* | *7 (35%)* | *0,41* | | *10* | *10 (100%)* | *4,94* |
|  | **2016** | | Any culture | 33 | 11 (33%) | 2,91 | | 0 | 0 | 0,00 | | 114 | 9 (8%) | 0,79 | | 23 | 11 (48%) | 35,60 | | 51 | 8 (16%) | 0,46 | | 31 | 26 (84%) | 12,69 |
|  |  | | *Blood culture* | *2* | *1 (50%)* | *0,26* | | *0* | *0* | *0,00* | | *0* | *0* | *0,00* | | *1* | *1 (100%)* | *3,24* | | *35* | *7 (20%)* | *0,40* | | *5* | *5 (100%)* | *2,44* |

Number of patients tested positive for the mentioned HRMO in one year, without specification for culture side. Abbreviations; EMC: Erasmus MC University Medical Centre in Rotterdam, The Netherlands. KSK: Kardinal Schwarzenberg Klinikum in Salzburg, Austria. VGS: Vienna General Hospital in Vienna, Austria. INMI: National Institute for Infectious Diseaes ‘Lazzaro Spallanzani’ in Rome, Italy. ERU: Erciyes University in Kayseri, Turkey. TGH: Tzaneio General Hospital in Piraeus, Greece. CPK: carbapenemase-producing *Klebsiella pneumonia*, CPPA: carbapenemase-producing *Pseudomonas aeruginosa*, VRE: vancomycin-resistant *Enterococcus faecium*. N.D.: No Data.

#### Additional file 5: Implemented infection prevention and control measures per hospital

| **General IPC measures*** | **EMC (Netherlands)** | | | **KSK (Austria)** | | | **VGS (Austria)** | | | **INMI (Italy)** | | | **ERU (Turkey)** | | | **TGH (Greece)** | | |
| --- | --- | --- | --- | --- | --- | --- | --- | --- | --- | --- | --- | --- | --- | --- | --- | --- | --- | --- |
| **Screening** |  | | |  | | |  | | |  | | |  | | |  | | |
| Always triage patients upon entry (max. 1) | 1 | | | 0 | | | 1 | | | 1 | | | 1 | | | 0 | | |
| Screen patients that were hospitalized <2 months for >24h in hospital abroad. (max. 1) | 0 | | | 1 | | | 0 | | | 0 | | | 0 | | | 0 | | |
| Screen patients that were hospitalized in hospitals with HRMO-outbreak. (max. 1) | 0 | | | 1 | | | 1 | | | 1 | | | 1 | | | 0 | | |
| Place high-risk patients in pre-emptive isolation. (max. 1) | 1 | | | 1 | | | 1 | | | 1 | | | 0 | | | 0 | | |
| Screening healthcare workers (max. 1) | 0 | | | 0 | | | 0 | | | 1 | | | 0 | | | 1 | | |
| **Isolation measures** |  | | |  | | |  | | |  | | |  | | |  | | |
| Isolation of patient who already have an isolation label in their electronic health record (max. 1) | 1 | | | 1 | | | 1 | | | 1 | | | 1 | | | 0 | | |
| Isolation of patient when triage shows that the patient has increased risk of HRMO (max. 1) | 1 | | | 1 | | | 0 | | | 1 | | | 1 | | | 0 | | |
| Isolation of patient with positive HRMO culture (max. 1) | 1 | | | 1 | | | 1 | | | 1 | | | 1 | | | 1 | | |
| **Cleaning and disinfection** |  | | |  | | |  | | |  | | |  | | |  | | |
| Cleaning isolation room, during hospitalization (max. 1) | 1 | | | 1 | | | 1 | | | 1 | | | 1 | | | 1 | | |
| Cleaning isolation room, after discharge (max. 1) | 1 | | | 1 | | | 1 | | | 1 | | | 1 | | | 1 | | |
| Daily cleaning of the isolation room (max. 1) | 1 | | | 1 | | | 1 | | | 1 | | | 1 | | | 1 | | |
| **HRMO-specific IPC measures** |  | | |  | | |  | | |  | | |  | | |  | | |
| **Type of HRMO** | **CPK** | **CPPA** | **VRE** | **CPK** | **CPPA** | **VRE** | **CPK** | **CPPA** | **VRE** | **CPK** | **CPPA** | **VRE** | **CPK** | **CPPA** | **VRE** | **CPK** | **CPPA** | **VRE** |
| Primary case - targeted screening on hospitalized patients | 1 | 1 | 1 | 1 | 1 | 1 | 1 | 1 | 1 | 0 | 0 | 0 | 1 | 1 | 1 | 0 | 0 | 0 |
| Primary case - targeted screening on discharged patients | 1 | 1 | 1 | 0 | 0 | 0 | 0 | 0 | 0 | 0 | 0 | 0 | 0 | 0 | 0 | 0 | 0 | 0 |
| Secondary case – targeted screening on hospitalized patients | 1 | 1 | 1 | 1 | 1 | 1 | 1 | 1 | 1 | 1 | 1 | 1 | 0 | 0 | 0 | 0 | 0 | 0 |
| Secondary case – targeted screening on discharged patients | 1 | 1 | 1 | 0 | 0 | 0 | 0 | 0 | 0 | 0 | 0 | 0 | 0 | 0 | 0 | 0 | 0 | 0 |
| Outbreak – targeted screening on hospitalized patients | 1 | 1 | 1 | 1 | 1 | 1 | 1 | 1 | 1 | 1 | 1 | 1 | 0 | 0 | 0 | 0 | 0 | 0 |
| Outbreak – targeted screening on discharged patients | 1 | 1 | 1 | 0 | 0 | 0 | 0 | 0 | 0 | 0 | 0 | 0 | 0 | 0 | 0 | 0 | 0 | 0 |
| Isolation label for HRMO-positive patients | 1 | 1 | 1 | 0 | 0 | 0 | 0 | 0 | 1 | 1 | 1 | 1 | 1 | 1 | 1 | 1 | 0 | 1 |
| Isolation (multi or single bedroom) of positive patient | 1 | 1 | 1 | 0 | 1 | 0 | 1 | 1 | 1 | 1 | 1 | 1 | 0 | 0 | 0 | 0 | 0 | 0 |
| Using non-sterile gloves | 1 | 1 | 1 | 1 | 1 | 1 | 1 | 1 | 1 | 1 | 1 | 1 | 1 | 1 | 1 | 0 | 0 | 0 |
| Using disposable gowns | 1 | 1 | 1 | 1 | 1 | 1 | 1 | 1 | 0 | 1 | 1 | 1 | 1 | 1 | 1 | 0 | 0 | 0 |
| Using caps | 0 | 0 | 0 | 0 | 0 | 0 | 1 | 1 | 1 | 0 | 0 | 0 | 0 | 0 | 0 | 0 | 0 | 0 |
| Using (surgical) masks | 0 | 0 | 0 | 0 | 0 | 0 | 1 | 1 | 1 | 0 | 0 | 0 | 0 | 0 | 0 | 0 | 0 | 0 |
| Performing molecular typing | 1 | 1 | 1 | 1 | 1 | 1 | 1 | 1 | 1 | 1 | 1 | 1 | 0 | 0 | 0 | 1 | 0 | 0 |
| Replacing separation curtains after discharge | 1 | 1 | 1 | 0 | 0 | 0 | 0 | 0 | 0 | 0 | 0 | 0 | 1 | 1 | 1 | 0 | 0 | 0 |
| Disposables in the isolation room are discarded after discharge | 1 | 1 | 1 | 1 | 0 | 1 | 1 | 1 | 1 | 1 | 1 | 1 | 1 | 1 | 1 | 1 | 1 | 1 |
| **Total points** | **EMC** | | | **KSK** | | | **VGS** | | | **INMI** | | | **ERU** | | | **TGH** | | |
| **Type of HRMO** | **CPK** | **CPPA** | **VRE** | **CPK** | **CPPA** | **VRE** | **CPK** | **CPPA** | **VRE** | **CPK** | **CPPA** | **VRE** | **CPK** | **CPPA** | **VRE** | **CPK** | **CPPA** | **VRE** |
| **Total** | **21** | **21** | **21** | **16** | **16** | **16** | **18** | **18** | **18** | **18** | **18** | **18** | **14** | **14** | **14** | **8** | **6** | **7** |

*Hospitals were only rewarded points when they reported a compliance of >90% to the IPC measure. Dichotomous yes/no questions: No = 0 points, Yes = 1 point. Abbreviations; EMC: Erasmus MC University Medical Centre in Rotterdam, The Netherlands. KSK: Kardinal Schwarzenberg Klinikum in Salzburg, Austria. VGS: Vienna General Hospital in Vienna, Austria. INMI: National Institute for Infectious Diseaes ‘Lazzaro Spallanzani’ in Rome, Italy. ERU: Erciyes University in Kayseri, Turkey. TGH: Tzaneio General Hospital in Piraeus, Greece. CPK: Carbapenemase producing *Klebsiella pneumonia*. CPPA: Carbapenemase producing *Pseudomonas aeruginosa.* VRE: vancomycin-resistant *Enterococcus faecium*. IPC: infection prevention and control. Max.: maximum. HRMO: highly resistant microorganisms.
